# Active template synthesis

**DOI:** 10.1039/d4cs00430b

**Published:** 2024-09-05

**Authors:** Romain Jamagne, Martin J. Power, Zhi-Hui Zhang, Germán Zango, Benjamin Gibber, David A. Leigh

**Affiliations:** a Department of Chemistry, University of Manchester Oxford Road Manchester M13 9PL UK david.leigh@manchester.ac.uk; b School of Chemistry and Molecular Engineering, East China Normal University Shanghai 200062 P. R. China

## Abstract

The active template synthesis of mechanically interlocked molecular architectures exploits the dual ability of various structural elements (metals or, in the case of metal-free active template synthesis, particular arrangements of functional groups) to serve as both a template for the organisation of building blocks and as a catalyst to facilitate the formation of covalent bonds between them. This enables the entwined or threaded intermediate structure to be covalently captured under kinetic control. Unlike classical passive template synthesis, the intercomponent interactions transiently used to promote the assembly typically do not ‘live on’ in the interlocked product, meaning that active template synthesis can be traceless and used for constructing mechanically interlocked molecules that do not feature strong binding interactions between the components. Since its introduction in 2006, active template synthesis has been used to prepare a variety of rotaxanes, catenanes and knots. Amongst the metal–ion-mediated versions of the strategy, the copper(i)-catalysed alkyne–azide cycloaddition (CuAAC) remains the most extensively used transformation, although a broad range of other catalytic reactions and transition metals also provide effective manifolds. In metal-free active template synthesis, the recent discovery of the acceleration of the reaction of primary amines with electrophiles through the cavity of crown ethers has proved effective for forming an array of rotaxanes without recognition elements, including compact rotaxane superbases, dissipatively assembled rotaxanes and molecular pumps. This Review details the active template concept, outlines its advantages and limitations for the synthesis of interlocked molecules, and charts the diverse set of reactions that have been used with this strategy to date. The application of active template synthesis in various domains is discussed, including molecular machinery, mechanical chirality, catalysis, molecular recognition and various aspects of materials science.

## Abstract 2

The active template synthesis of rotaxanes, catenanes and knots exploits the dual ability of metals, or particular arrangements of functional groups, to serve as both a template and a catalyst to assemble and covalently capture interlocked molecules.

## Graphical abstract

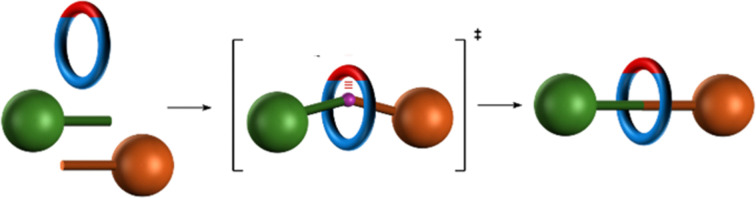

## Introduction

**Fig. 1:**
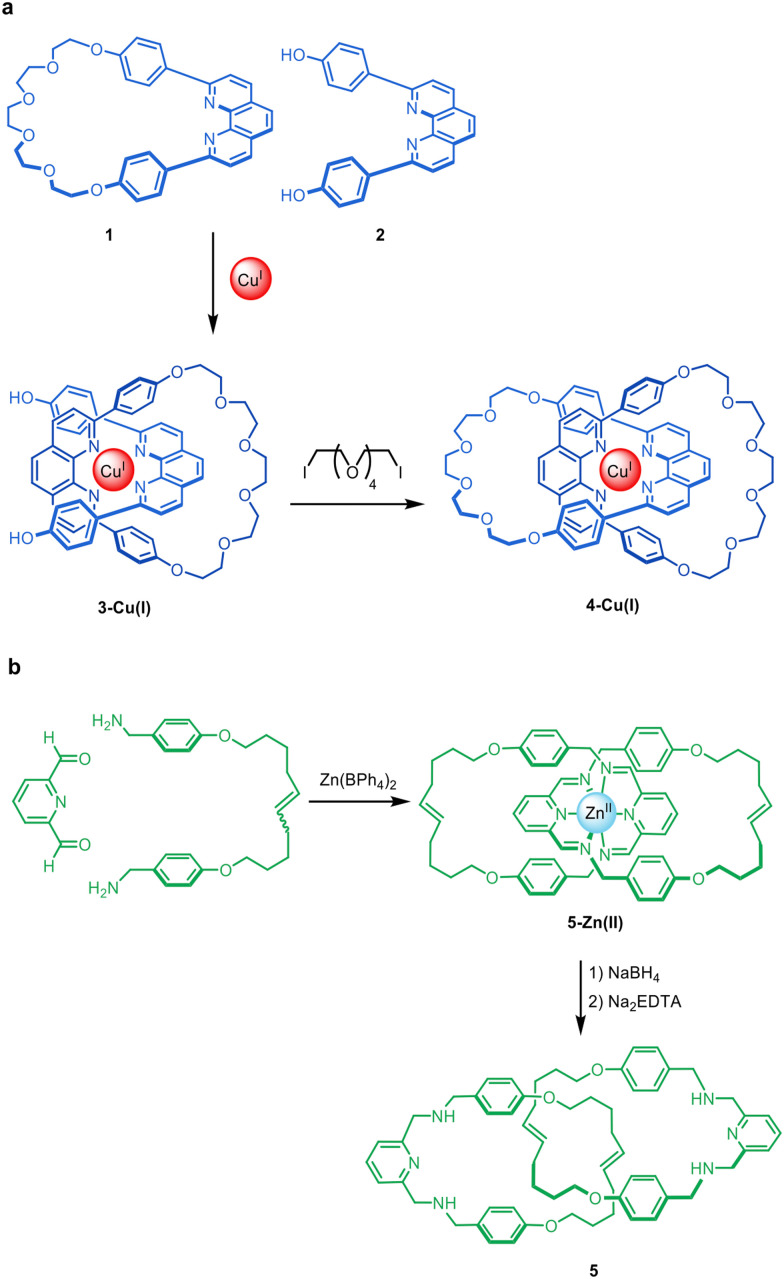
Classic examples of the passive metal template synthesis of interlocked molecules. (a) The Sauvage group's synthesis of catenane 4-Cu(i) in two sequential steps: coordination of the bidentate ligands to the metal in a tetrahedral geometry, followed by capturing of the threaded structure through covalent bond formation.^7^ (b) The Leigh group's synthesis of benzylic imine catenanes by imine formation about an octahedral Zn(ii) template, followed by reduction of the imines and extraction of the metal ions to give wholly organic catenane 5.^9^ Although the Zn(ii) ions promote formation of the imine bonds in addition to holding the ligands in place, the metal cannot be removed from the imine catenate without a subsequent reduction step to stabilise the covalent framework in the absence of the metal. The two-step synthesis of 5 is therefore an example of passive metal template synthesis.

The synthesis of mechanically interlocked molecules (*e.g.* catenanes, rotaxanes and knots) has been explored since the middle of the 20th century.^1,2^ Early methods for their synthesis, such as the statistical approach of Wasserman^3,4^ (catenanes) and Harrison and Harrison (rotaxanes)^5^ or the directed synthesis strategies of Lüttringhaus and Schill,^6^ resulted in very low yields and/or required long synthetic schemes. In 1983, Sauvage and co-workers introduced a sea change in synthetic approaches to interlocked molecules by combining macrocycle 1 and diol 2 with Cu(i) to form complex 3-Cu(i), which subsequently underwent macrocyclisation of the threaded phenanthroline unit with a oligoethylene glycol chain to form metallo-catenane 4-Cu(i) in 42% yield (Fig. 1a).^7,8^ The key feature of this synthesis was the use of the metal ion as a template to hold the organic components in a threaded arrangement such that connecting the end groups together resulted in a mechanically linked molecule (Fig. 1).

**Fig. 2:**
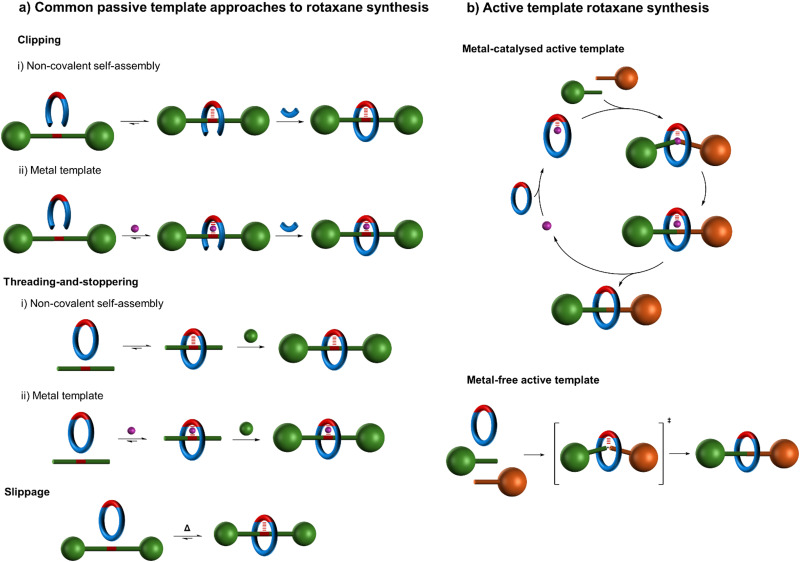
Schematic representation of (a) various common passive template strategies for rotaxane synthesis^18–20^ and (b) active template synthesis of rotaxanes.^19,20,22^ (a) The ‘clipping’ and ‘threading-and-stoppering’ approaches involve thermodynamically driven assembly processes followed by capturing of the interlocked structure by covalent bond formation.^18^ The ‘slipping’ or ‘slippage’ approach^18^ involves the macrocycle passing over the stoppers at an elevated temperature; at lower temperatures the macrocycle is then kinetically trapped on the axle. As a covalent bond does not need to be broken to disassemble such a threaded structure, such supramolecular assemblies are better described as kinetically stable pseudo-rotaxanes rather than as rotaxanes (see footnote 194 in ref. 21). (b) Active template synthesis is kinetically driven; component assembly and the covalent capture of the interlocked structure occur contemporaneously.

Since this seminal work, numerous strategies for positioning molecular components for interlocking have been developed, utilising diverse metal coordination modes and geometries^10–12^ and/or noncovalent interactions based on aromatic stacking interactions,^13,14^ hydrogen bonding,^15^ and/or hydrophobic effects^16,17^ (Fig. 2).^18^ The classic metal template approach^7,9–12^ to interlocked molecules involves two distinct processes which are generally accomplished step-wise (Fig. 2a):

(i) Component positioning: in which the well-defined coordination geometry of the metal is used to arrange ligands such that joining them together favours the formation of a mechanically interlocked structure.

(ii) Component connecting: a subsequent step in which the threaded arrangement present in the supramolecular complex is captured through one or more covalent bond-forming reactions.

As the component positioning step only exploits the coordination geometry of the metal ion, rather than any ability of the metal to promote the formation of covalent bonds that persist once the metal has been removed, this approach can be categorised as ‘passive’ metal template synthesis.^10^ In contrast, ‘active’ metal template synthesis^19,20,22^ (Fig. 2b) proceeds in a single step that exploits both the coordination geometry of a metal (in acting as a template to organise ligands into a threaded or entwined intermediate), and the metal's ability to catalyse covalent bond-forming reactions (in order to covalently capture the interlocked structure).

Passive and active template methodologies diverge in the principles that underpin them (Fig. 2).^19^ The passive template approach generally involves the formation of a thermodynamically favourable threaded or entwined intermediate complex (Fig. 2a).^18^ This minimum energy arrangement of building blocks is subsequently captured, typically by macrocyclisation or stoppering, in a second, bond-forming, process. In contrast, active template synthesis (Fig. 2b) does not involve a stable intermediate, but rather depends upon a catalysed bond-forming reaction occurring faster through a macrocyclic cavity than outside of it, irrespective of whether any attractive noncovalent interactions between the components are present in the final product.^19,20^ The active template process is thus kinetically driven and offers the prospect of assembling thermodynamically unfavourable interlocked structures.

Active template synthesis offers a number of distinctive features and potential advantages compared to passive template strategies:

(i) The absence of recognition motifs between the components of the final product provides an opportunity for the traceless synthesis of so-called ‘impossible’ rotaxanes.^23–26^

(ii) Sub-stoichiometric amounts of the metal can often be used,^22,27^ which turn over during their catalysis of the covalent bond-forming reaction(s).

(iii) The formation of a sterically shielded threaded structure can kinetically stabilise otherwise transient intermediates, aiding the elucidation of reaction mechanisms.^28^

(iv) The kinetically driven nature of active template synthesis allows for the formation of kinetically stable (because of the mechanical bond), but thermodynamically unstable, interlocked structures.^19,20^

## Active template Cu-catalysed alkyne–azide cycloaddition

### The concept of catalytically forming mechanical bonds

**Fig. 3:**
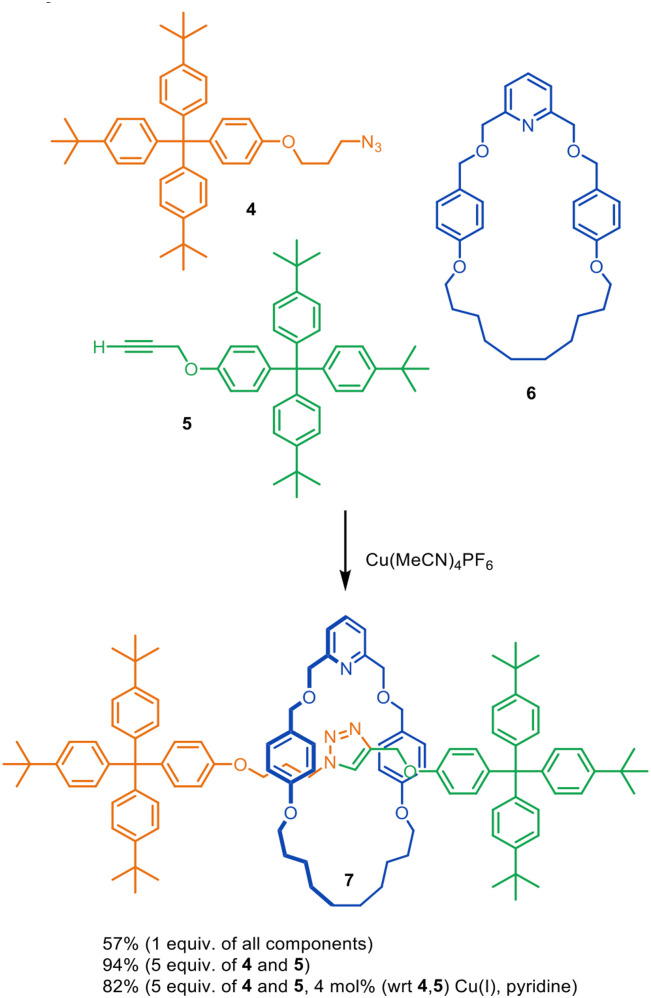
Introduction of active metal template synthesis by Leigh and co-workers.^22^

The concept of combining metal catalysis with template organisation for the assembly of interlocked molecules was introduced by Leigh and co-workers in 2006.^22^ A macrocycle pyridine group was used to bind Cu(i) within the macrocyclic cavity such that transiently coordinated azide and alkyne ligands were forced to attach to the tetrahedral Cu(i) centre through opposite faces of the macrocycle. The endotopic Cu(i) ion (and a second Cu(i) ion^28^) then promotes the Cu-catalysed alkyne–azide cycloaddition (CuAAC) of the attached ligands to form the threaded axle. The reaction of azide 4, alkyne 5, pyridine macrocycle 6 and Cu(MeCN)_4_PF_6_ produced [2]rotaxane 7 in up to 94% yield (Fig. 3).^22^ A sub-stoichiometric amount of the Cu(i) catalyst could be used, with loadings as low as 4 mol% still resulting in 82% yield of rotaxane 7. A 2007 study investigated the influence of macrocycle structure, and found that a strongly coordinating mono- or bidentate motif (pyridine or bypyridine) and minimal steric bulk or rigidity around the metal centre were most effective for rotaxane formation.^28^

In 2011, the Goldup group found that smaller bipyridine macrocycles gave higher [2]rotaxanes yields, particularly with short axles.^29^ The Crowley group subsequently demonstrated that for *exo*-alcohol-functionalised pyridyl macrocycles, even smaller macrocycles could be used,^30^ including 24-membered rings which begin to approach the limit possible^31^ for accommodating the thickness of a threaded saturated hydrocarbon chain.

### Insights into the mechanism of CuAAC catalysis

The pathway of CuAAC catalysis begins with Cu(i) acetylide formation, followed by [3+2] cycloaddition with an azide, creating a Cu(i)-triazolizide species as a key intermediate.^32^

**Fig. 4:**
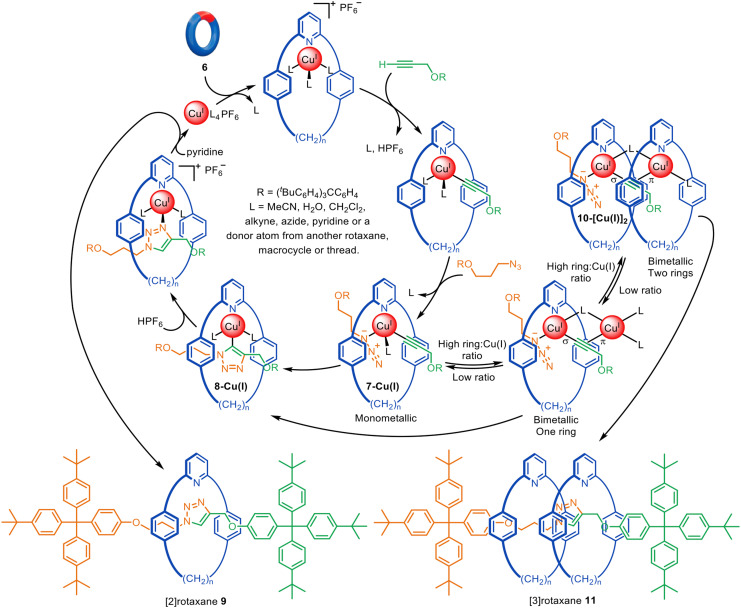
Mechanism of the active template CuAAC reaction leading to [2]- or [3]rotaxanes depending on the ratio of macrocycle to Cu(i).^28^

In the early-2000s various intermediates for the CuAAC reaction had been postulated in experimental and computational studies.^33–35^ However, unambiguous evidence for various intermediates, and in particular whether one or two Cu(i) atoms were involved, remained unclear. In 2007 Leigh and co-workers reported unexpected [3]rotaxane formation during active metal template synthesis with a pyridine macrocycle (Fig. 4).^28^ The trapping of two rings threaded onto an axle produced by the formation of one triazole group, using macrocycles that could clearly bind to only one metal ion each, provided clear experimental evidence that two distinct Cu(i) atoms are involved in catalysing the [3+2] cycloaddition, at least with this type of ligand system.

In the proposed reaction pathway (Fig. 4), macrocycle 6 and the half-axle components bind to Cu(i) to form 7-Cu(i). This intermediate then undergoes the Cu(i)-catalysed 1,3-cycloaddition leading to interlocked complex 8-Cu(i) and, ultimately, metal-free [2]rotaxane 9. Alternatively, the second metal ion of 10-[Cu(i)]_2_ can be bound to another macrocycle prior to the azide–alkyne cycloaddition occurring, leading to [3]rotaxane 11. The formation of [3]rotaxane is enhanced by increasing the macrocycle : Cu(i) ratio, consistent with this mechanistic interpretation. Goldup and coworkers later found that high temperatures, non-coordinating solvents and larger macrocycles can also increase the amount of [3]rotaxane formed.^36^

Goldup and coworkers have isolated a Cu(i)-triazolizide rotaxane complex from an active template CuAAC reaction.^37^ The complex was remarkably stable, even under aqueous conditions, with the tightly threaded macrocycle sterically inhibiting protonation of the Cu–C bond. This was the first triazolide intermediate isolated from a CuAAC reaction.

These findings illustrate well how mechanical bonding can provide mechanistic insights into the catalysis manifolds used for active template synthesis.

### CuAAC-mediated active metal template synthesis of catenanes and knots

CuAAC-mediated active template synthesis has been used to synthesise a broad range of rotaxanes, including [2]rotaxanes *via* either threading^22,28^ or clipping,^38^ heterocircuit [3]rotaxanes,^39^ doubly threaded [3]rotaxanes^40^ and triply threaded [4]rotaxanes.^41^ Its application to other topologies has been somewhat less explored. In 2009, the Leigh group used CuAAC active template synthesis to produce homocircuit and heterocircuit [2]catenanes in up to 53% yield.^42^ The Goldup group subsequently improved the scope and yields, making catenanes with smaller macrocycles.^43^ Their improvement is particularly notable as the earlier active template syntheses of catenanes^42^ required high dilution and long reaction times (typically 1–7 days), whereas Goldup's system gave yields of up to 98% in 4 hours. Otsuka and co-workers have reported the synthesis of [3]catenanes using CuAAC active template rotaxane formation followed by macrocyclisation of the bis(2,2,6,6-tetramethyl-piperidin-1-yl) disulfide stoppers into a catenane.^44^

**Fig. 5:**
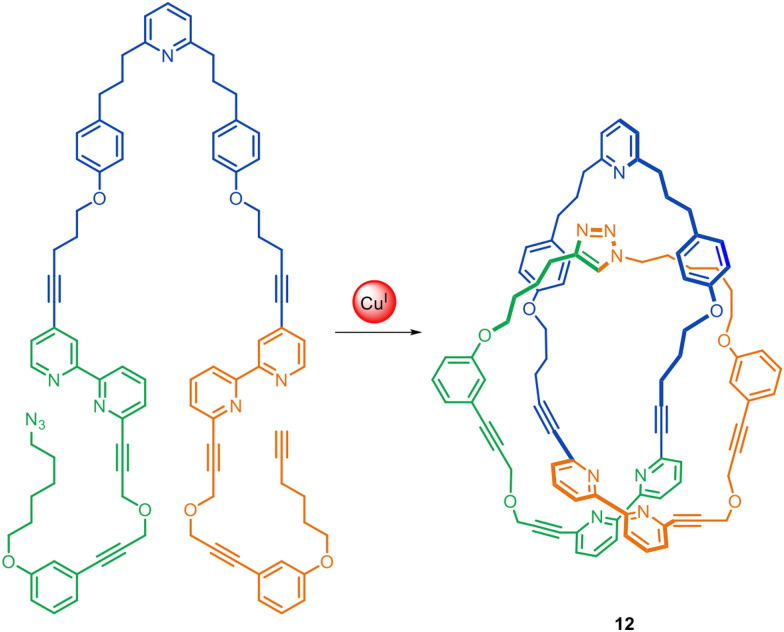
Molecular trefoil knot 12 synthesised through active template CuAAC synthesis through the macrocyclic loop formed by Cu(i)-coordination to the two bipyridine groups.^45^

Leigh and co-workers have assembled a trefoil knot 12 using a combination of passive and active template processes (Fig. 5).^45^ The trefoil knot is formed in 24% yield from a single molecular strand, using one Cu(i) ion to coordinate to both bipyridine units, forming a loop in the strand, while a second Cu(i) ion acts as the active template site, catalysing the CuAAC reaction of the alkyne and azide termini through the loop.^45^

## Active template Glaser–Hay oxidative homocoupling of terminal alkynes

**Fig. 6:**
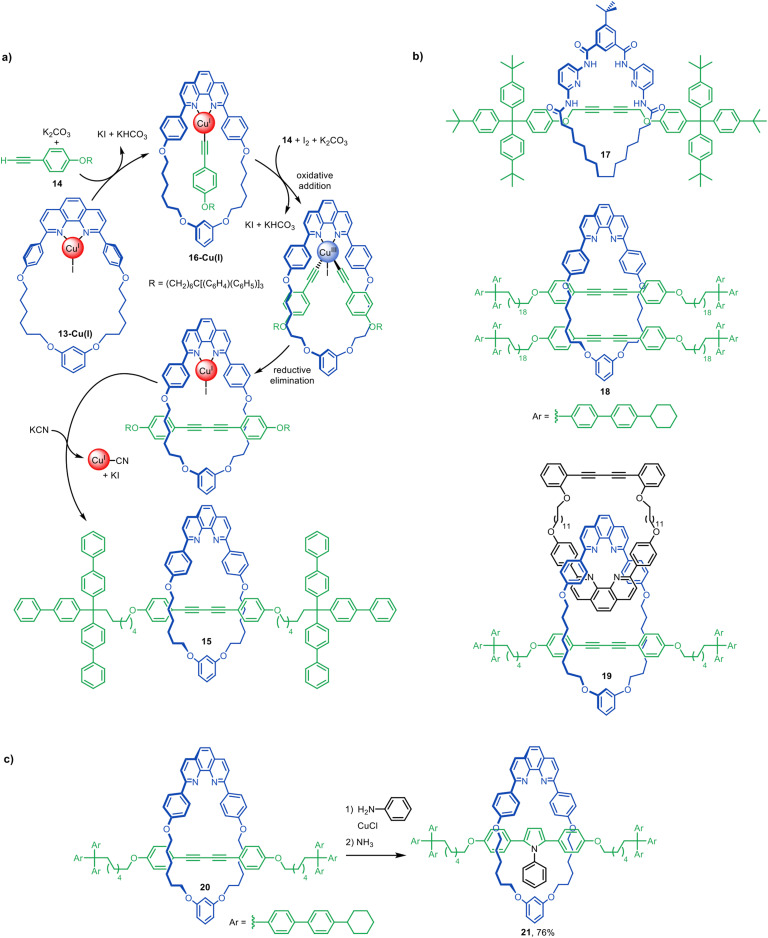
(a) Proposed mechanism for the active template synthesis of [2]rotaxanes through the Cu(i)-mediated Glaser–Hay homocoupling of terminal alkynes.^46^ (b) Selected examples of interlocked structures synthesised by active template Glaser couplings.^47–49^ (c) Saito's post-assembly modification a diyne rotaxane to an aryl pyrrole rotaxane.^50^

The Leigh group's introduction of the active template synthesis concept^22^ (Fig. 3) was followed about a year later by Saito and co-workers use of stoichiometric quantities of a preformed macrocyclic Cu(i)-phenanthroline complex, 13-Cu(i), to promote the Glaser–Hay oxidative homocoupling of alkyne 14 (Fig. 6a).^46^ This generated [2]rotaxane 15 in 72% yield. The reaction likely proceeds through the pathway shown in Fig. 6a. First, alkyne 14 adds to 13-Cu(i) to form Cu(i)-acetylide intermediate 16-Cu(i). The Cu(i) centre is then oxidised by I_2_ and a second alkyne adds to the resulting Cu(iii) species (or possibly a bimetallic Cu(ii) intermediate) on the opposite face of the macrocycle. Reductive elimination generates the covalent bond that captures the threaded structure. The Cu(i) is then removed with KCN to give metal-free [2]rotaxane 15.

Active template Glaser and Glaser-type couplings have subsequently been used to synthesise a variety of interlocked molecules, including [2]rotaxanes,^47,50–64^ a [2]catenane,^65^ and doubly threaded [3]rotaxanes such as 18 (Fig. 6b).^48,66^ The approach can be combined with passive template approaches to create more elaborate architectures such as 19.^49^ Its suitability for forming π-conjugated rotaxanes has been utilised to synthesise an array of ‘molecular wires’ (see section on sheathed ‘molecular wires’).

Although most active template Glaser couplings use Cu(i)-phenanthroline complexes, an exception is [2]rotaxane 17 (Fig. 6b), synthesised from a macrocyclic Hamilton-type receptor, albeit in modest (9%) yield.^67^

Saito and co-workers have investigated the effects of macrocycle size, axle length and stoichiometry on the yields of singly threaded [2]- and doubly threaded [3]rotaxanes made *via* active template Glaser couplings. The ratio of [3]rotaxane to [2]rotaxane was increased using larger macrocycles and longer thread building blocks.^48^

Interlocked molecules prepared by Glaser couplings are readily modified by reaction of the diyne, increasing the diversity of structures accessible using this active template reaction. Saito and co-workers described the post-modification of rotaxane 20 to [2]rotaxane 21 bearing an aryl pyrrole moiety in the axle (Fig. 6c).^50^ This allowed the shuttling dynamics of rotaxanes with different levels of steric bulk in the axle to be studied,^54^ as well as chiral HPLC separation of mechanically planar chiral rotaxanes with rotationally asymmetric macrocycles.^53^

## Active template Cadiot–Chodkiewicz heterocoupling of terminal alkynes with alkyne halides

Active template Glaser–Hay homocoupling reactions result in rotaxanes with a diyne axle with mirror plane symmetry. Unsymmetrical diyne axle rotaxanes are accessible through the active template Cadiot–Chodkiewicz heterocoupling of alkynes and alkyne halides. An unsymmetrical axle is useful for switchable rotaxane-based molecular shuttles, pumps and other types of molecular devices.

**Fig. 7:**
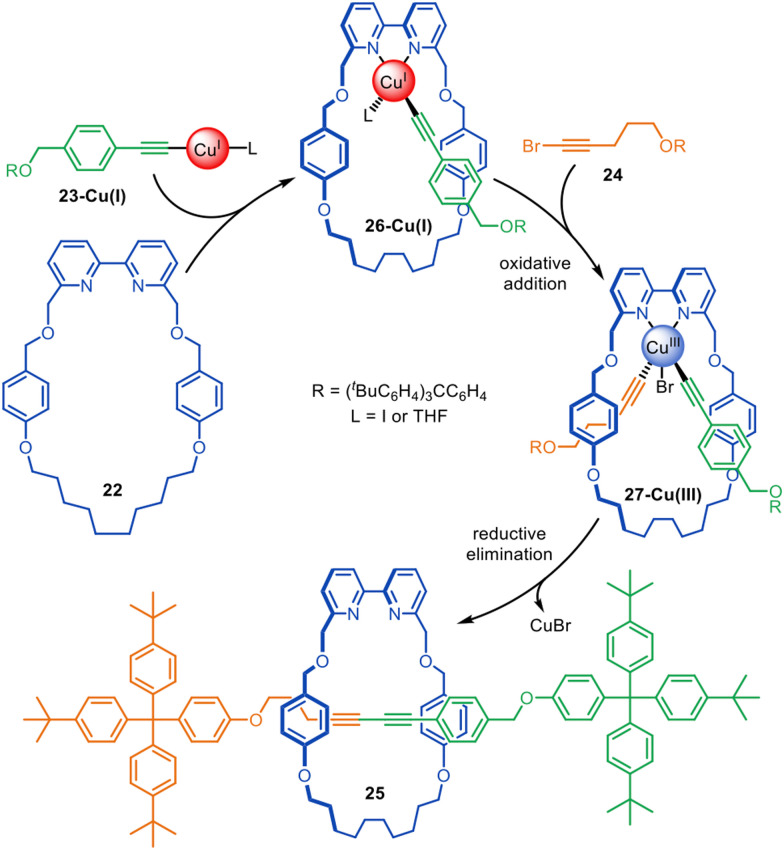
Proposed mechanism for the active template synthesis of [2]rotaxanes through the Cu(i)-mediated Cadiot–Chodkiewicz heterocoupling of terminal alkynes and alkyne halides.^67^

The Cadiot–Chodkiewicz active template synthesis of [2]rotaxanes was introduced by Leigh and co-workers in 2008 (Fig. 7).^67^ This reaction can be particularly sensitive to the order in which the building blocks, reagents and catalyst are combined: mixing a terminal alkyne, alkyne halide and bipyridine macrocycle 22 with an amine base and CuI resulted in a mixture of desired heterocoupling and undesired homocoupling products.^67^ In contrast, preforming copper acetylide 23-Cu(i), followed by subsequent addition of the macrocycle and alkyne halide 24 increased the rotaxane yield up to 85% with almost exclusive selectivity for the heterocoupled [2]rotaxane 25. The mechanism appears to proceed through the formation of Cu(i)-acetylide complex 26-Cu(i), which undergoes oxidative addition of 24 through the opposite face of the macrocycle to form 27-Cu(iii). Reductive elimination affords the heterocoupled product, rotaxane 25. The strategy was later extended to the active template synthesis of heterocircuit [2]catenanes.^42^

The Cadiot–Chodkiewicz active template reaction has been used for the synthesis of a variety of interlocked structures,^68–71^ often with better yields than the comparative Glaser couplings or enabling the formation of rotaxanes that were not accessible through alkyne homocouplings.^68^ It has proved particularly well-suited for the synthesis of polyyne and cumulene [2]rotaxanes, which generally show improved stability over their non-interlocked equivalents (see section on sheathed ‘molecular wires’).

**Fig. 8:**
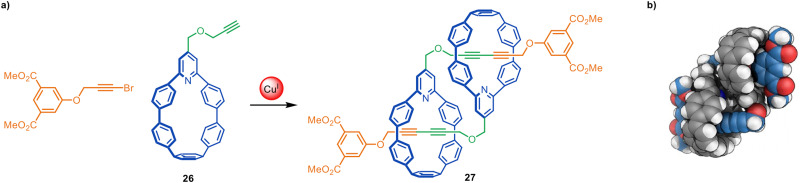
(a) Jasti's synthesis of a [*c*2]daisy-chain rotaxane 27 from an active template Cadiot–Chodkiewicz reaction. (b) Space-filling representation of 27.^69^

Jasti and coworkers have made an impressive range of fluorescent ‘nanohoop’ [2]rotaxanes and [*c*2]daisy-chain rotaxanes through Cadiot–Chodkiewicz active template synthesis. The structures feature a shape-persistent macrocyclic belt containing a 2,6-connected pyridine residue (*e.g.*26) to endotopically coordinate the metal that promotes rotaxane formation. The [*c*2]daisy-chain rotaxane 27 has a relatively compact structure, apparent from the space-filling representation of the X-ray structure (Fig. 8).^69,70^

## Other copper-mediated active metal template reactions

**Fig. 9:**
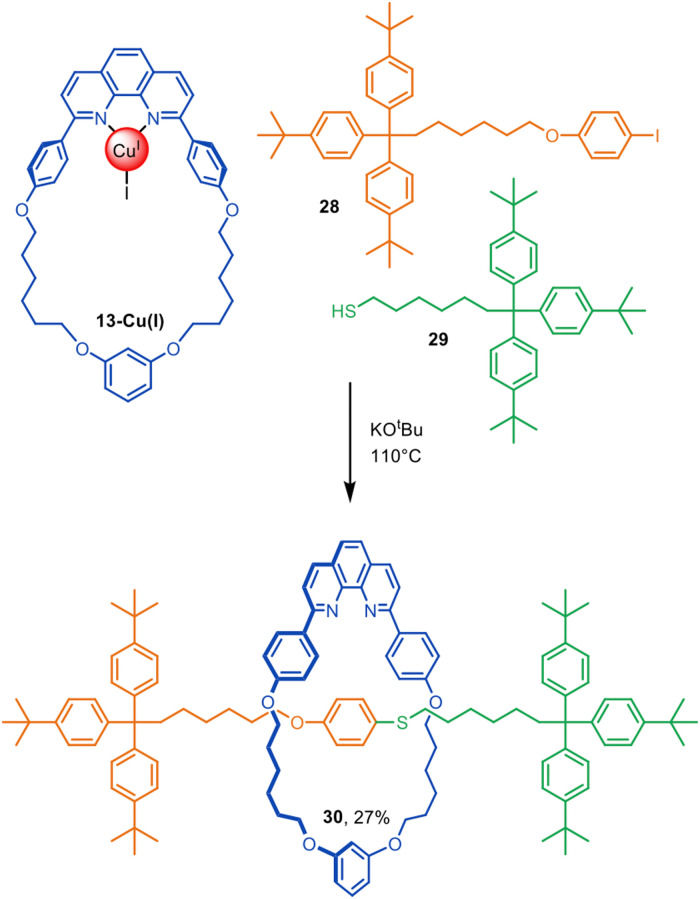
Saito's active template synthesis of a [2]rotaxane by Ullmann C–S coupling of a thiol and aryl iodide.^46^

In addition to using 13-Cu(i) in Glaser–Hay active template synthesis, Saito and co-workers used the phenanthroline complex in a copper-mediated Ullmann C–S heterocoupling of aryl iodide 28 and thiol 29 (10-fold excess of each) to give [2]rotaxane 30 in a modest (27%) yield (Fig. 9).^46^

**Fig. 10:**
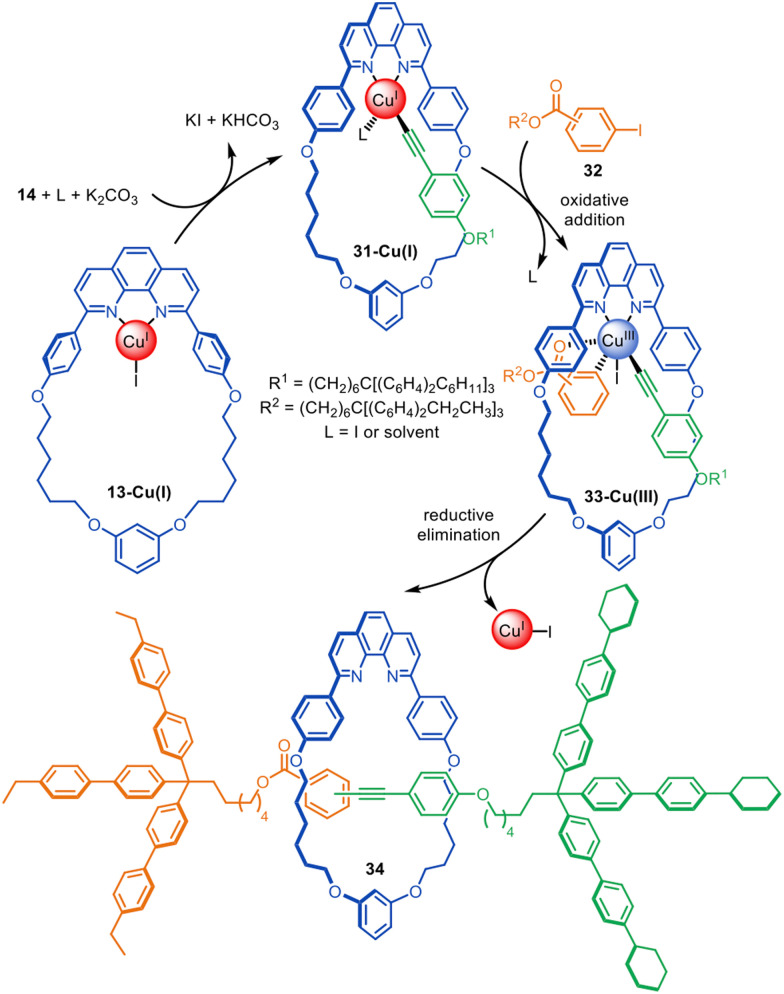
Saito's active template synthesis of [2]rotaxanes through the Cu(i)-mediated Sonogashira-type coupling of terminal alkynes and aryl iodides.^72^

The same Cu(i)-phenanthroline macrocycle has also been used in a Sonogashira-type active template heterocoupling of aryl iodides and terminal alkynes, generating rotaxanes in up to 52% yield (Fig. 10).^72^ Concurrent oxidative homocoupling of the terminal alkynes occurs in such reactions and so the corresponding diyne rotaxanes were formed as side-products in each case. The mechanism is presumed to proceed *via* the formation of Cu(i)-acetylide 31-Cu(i), followed by oxidative addition of aryl iodide 32 to form Cu(iii)-complex 33-Cu(iii). The latter undergoes reductive elimination through the macrocycle cavity to form [2]rotaxane 34. The best yields and selectivity for the heterocoupling product were obtained using the *ortho*-isomer of 32.

Leigh and co-workers have used Cu(i) catalysis in the active template Goldberg amidation of aryl halides to form [2]rotaxanes with chiral *C*_2_-symmetric cyclohexyldiamine macrocycles.^73^ The mechanism is similar to the active template Cu(i)-mediated Sonogashira-type coupling (Fig. 10), with an aryl amide rather than an alkyne undergoing ligation with 21-Cu(i). The resulting chiral rotaxanes were subsequently investigated as ligands for enantioselective metal catalysis (see section on asymmetric, diastereoselective and other types of catalysts).

## Other transition-metal-mediated active template reactions

Copper-catalysed reactions have proved to be particularly successful and robust catalytic manifolds for active template synthesis. This is because copper catalysis is often promoted by particular ligand types (which can form the basis of a suitable macrocycle) and because of the well-defined 3D coordination geometries of Cu(i) and Cu(iii) which aid ligation of the axle building blocks through opposite faces of the macrocycle. However, copper is somewhat limited in its ability to activate sp^3^-hybridised carbons compared to other metals such as palladium,^74^ nickel,^75^ rhodium^76^ and ruthenium.^77^ In the next sections we give examples of how reactions catalysed by these, and a number of other transition metals, have also proved amenable for active template synthesis.

### Palladium-catalysed active template synthesis

Palladium-catalysed cross-coupling transformations are extensively employed in organic synthesis for the construction of C–C bonds. Although Pd(ii) has a 2D square planar coordination geometry, which is poorly suited for endotopic binding within a macrocyclic cavity compared to the 3D tetrahedral geometry of Cu(i), palladium offers high catalytic activity in numerous reactions. Attempts by the Leigh group to use cross-couplings that feature transient Pd(0) in the catalytic cycle proved unsuccessful for active template synthesis, apparently because of the poor coordination of Pd(0) leading to detachment of the metal from various macrocycle types during key steps of the catalytic cycle.^27^

**Fig. 11:**
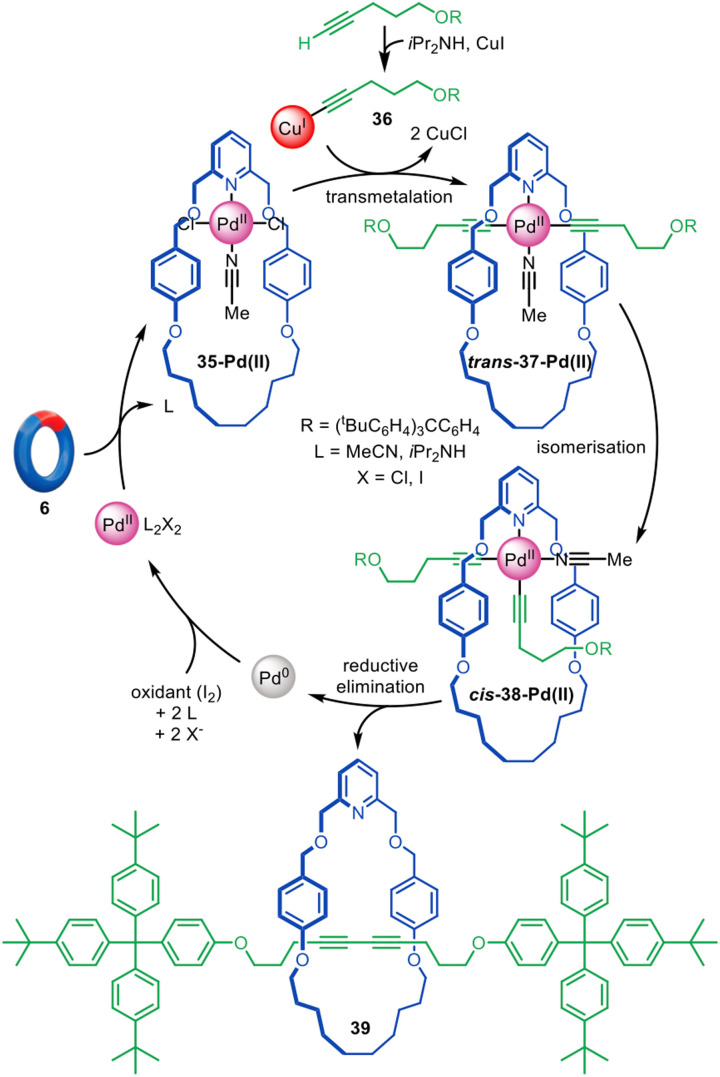
Active template synthesis of [2]rotaxanes through the Pd(ii)-mediated oxidative homocoupling of terminal alkynes.^84^

Leaning on previously reported Pd(ii)-mediated passive template syntheses of rotaxanes^78–81^ and catenanes for the macrocyclic ligand design,^82,83^ Leigh and co-workers showed that, despite its square planar coordination geometry, Pd(ii) can be a suitable metal for the active template synthesis of [2]rotaxanes through alkyne homocoupling (Fig. 11).^84^ The reaction proceeds with the replacement of the *trans*-chloride ligands of 35-Pd(ii) by the Cu(i)-acetylide unit 36 to form a threaded *trans*-37-Pd(ii) complex. Isomerisation to *cis*-38-Pd(ii) occurs without breaking the Pd–acetylide bond, preserving the threaded architecture. Reductive elimination occurs from the *cis* configuration to yield diyne [2]rotaxane 39 after decomplexation of the weakly coordinating Pd(0). Only a catalytic amount of Pd(ii) (5 mol%) is required, together with I_2_ or O_2_ to reoxidise the Pd(0) formed *in situ*, giving rotaxane yields up to 90%.

**Fig. 12:**
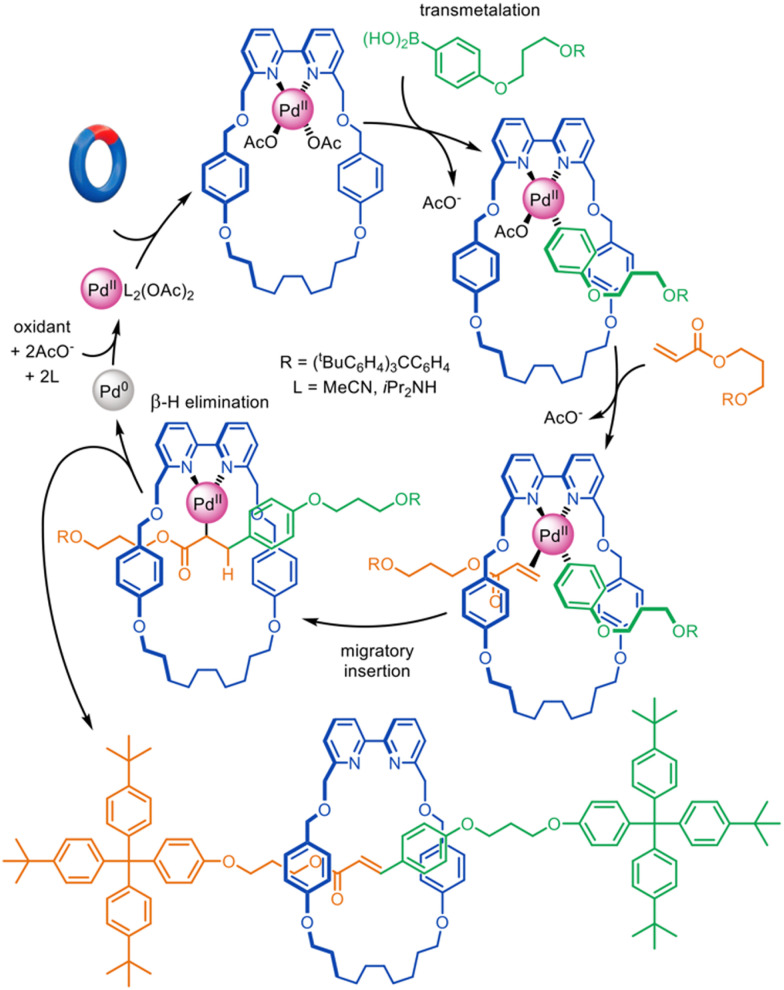
Active template synthesis of [2]rotaxanes through Pd(ii)-mediated oxidative Heck cross-coupling.^27^

The oxidative Heck cross-coupling reaction^85^ is a variant of palladium cross-couplings that does not feature Pd(0) in the mechanism. Accordingly, it is more suitable as the basis for active template synthesis. Leigh and co-workers demonstrated its efficacy in the synthesis of a variety of [2]rotaxanes using a bipyridine macrocycle and boronic acid and electron-poor alkene axle building blocks (Fig. 12).^27^ The catalytic loading could be as low as 1 mol% while still furnishing a rotaxane yield of 66%, the largest catalytic turnover number reported to date for an active template reaction.

The examples in Fig. 11 and 12 demonstrate the significant potential of palladium for active template synthesis, particularly if macrocyclic ligands can be found that can overcome the lability of Pd(0) so that it remains coordinated to the components during various important steps in the mechanism.

**Fig. 13:**
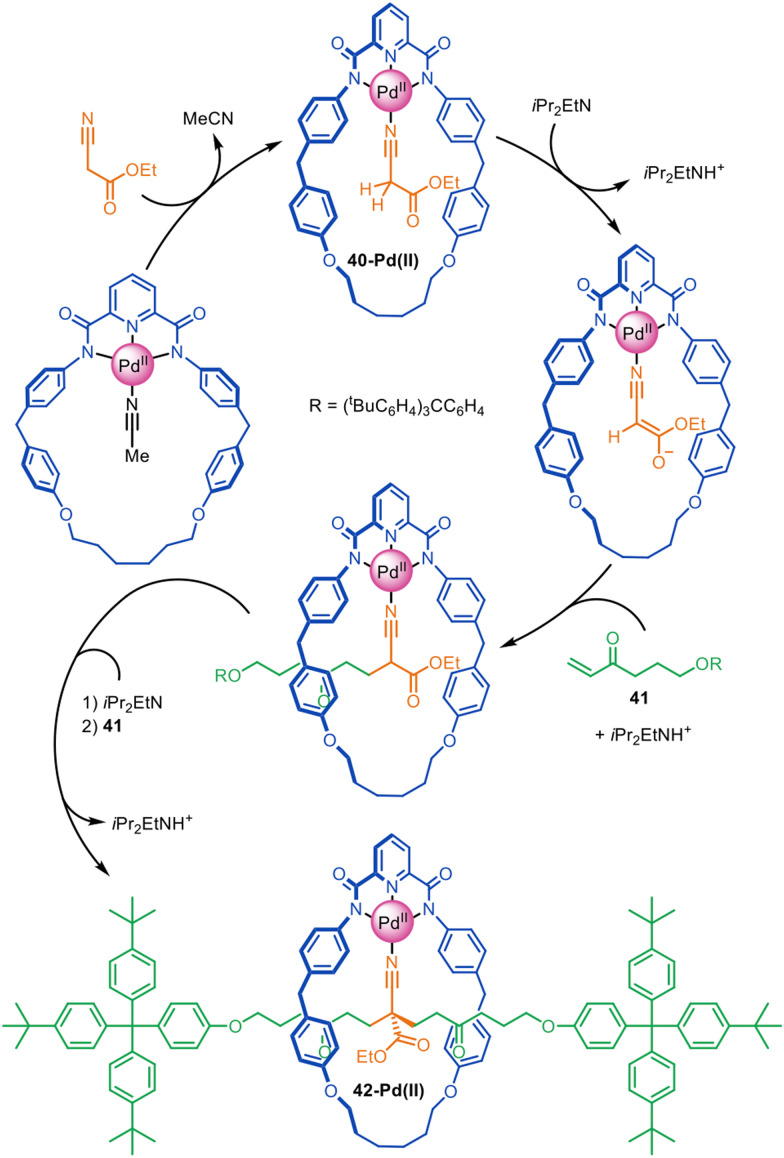
Active template synthesis of [2]rotaxanes through successive Pd(ii)-mediated Michael additions of four components.^86^

Very high yielding four-component synthesis of rotaxanes by successive Pd(ii)-promoted Michael additions of α-cyano esters to alkyl vinyl ketones has been reported using tridentate pyrroloimidazolone or bisoxazoline pincer macrocycles (Fig. 13).^86^ The formation of complex 40-Pd(ii) enables two consecutive 1,4-conjugate additions to α,β-unsaturated ketone 41 from opposite macrocycle faces to quantitatively form [2]rotaxane 42-Pd(ii). In this example the template interactions between thread and macrocycle remain in the rotaxane product, a rare feature for active template synthesis. Accordingly, the intercomponent interactions were used to good effect for the synthesis of rotaxanes with well-defined switching of the macrocycle position on the axle (molecular shuttles).

### Nickel-mediated active template synthesis

An unusual dual Ni-/Cu-mediated alkyne homocoupling active template reaction has been reported.^87^ The system uses an octahedral Ni(ii) macrocyclic complex followed by addition of CuI which results in reductive elimination of the half-axle ligands to yield [2]rotaxanes. The synthesis was high yielding (up to 95%), although stoichiometric quantities of NiCl_2_ and CuI are required.

Active template synthesis that deletes atoms from the building blocks while joining them to form a hydrocarbon region of a threaded axle is a particularly appealing target because it is completely traceless. The main obstacle to doing so lies in achieving effective and catalytic formation of C(sp^3^)–C(sp^3^) bonds using organometallic chemistry. This is generally difficult due to slow rates of oxidative addition of metal centres to C(sp^3^)–heteroatom bonds, concomitant with facile β-hydride elimination of the resulting organometallic intermediate.^88–91^ Most successful C(sp^3^)–C(sp^3^) coupling protocols involve alkyl halides activated towards oxidative addition and lacking β-hydrogens.^92,93^

**Fig. 14:**
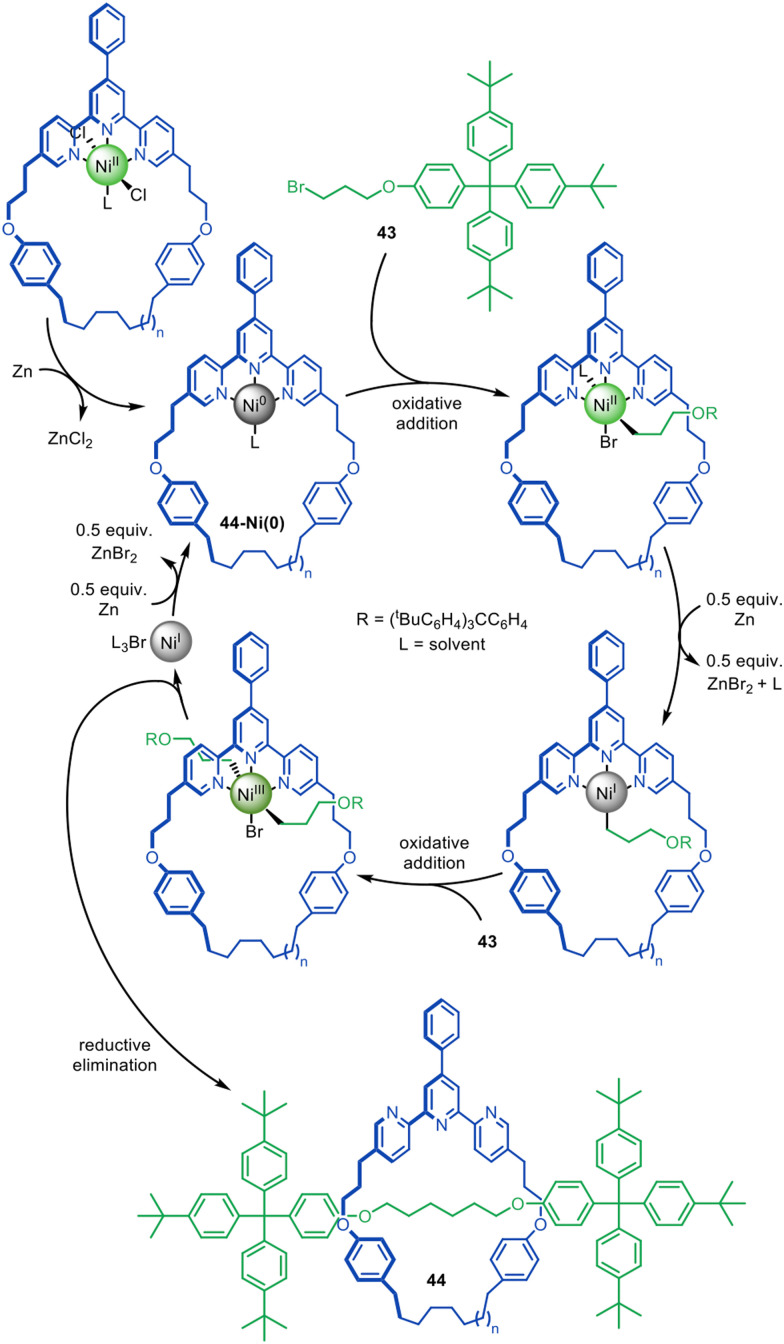
Active template synthesis of traceless [2]rotaxanes through the Ni(0)-mediated homocoupling of unactivated alkyl bromides.^99^

Adapting the work of Fu and co-workers on the coupling of unactivated alkyl halides,^94–98^ the active template synthesis of alkyl chain axle-region rotaxanes was achieved by Leigh and co-workers through the Ni(0)-catalysed C(sp^3^)–C(sp^3^) homocoupling of bromoalkanes (Fig. 14).^99^ The reaction was initially thought to likely proceed through a Negishi-type mechanism,^91^ however mechanistic studies indicate a pathway similar to the Ni-mediated coupling of aryl halides.^100^ Oxidative addition of alkyl bromide 43 to 44-Ni(0) is followed by the one-electron reduction of Ni(ii), allowing a second oxidative addition of 43, ligating the axle building blocks through opposite faces of the macrocycle. Reductive elimination then captures threaded architecture 44, while Zn(0) reduces the resulting Ni(i) species to catalytically active Ni(0).

Saito and co-workers have carried out the cross-coupling of iodoarenes mediated by a dibenzodihydrophenanthroline–Ni complex.^101^ This was used for the synthesis of [2]rotaxanes with up to ten *para*-phenylene units in the axle.

**Fig. 15:**
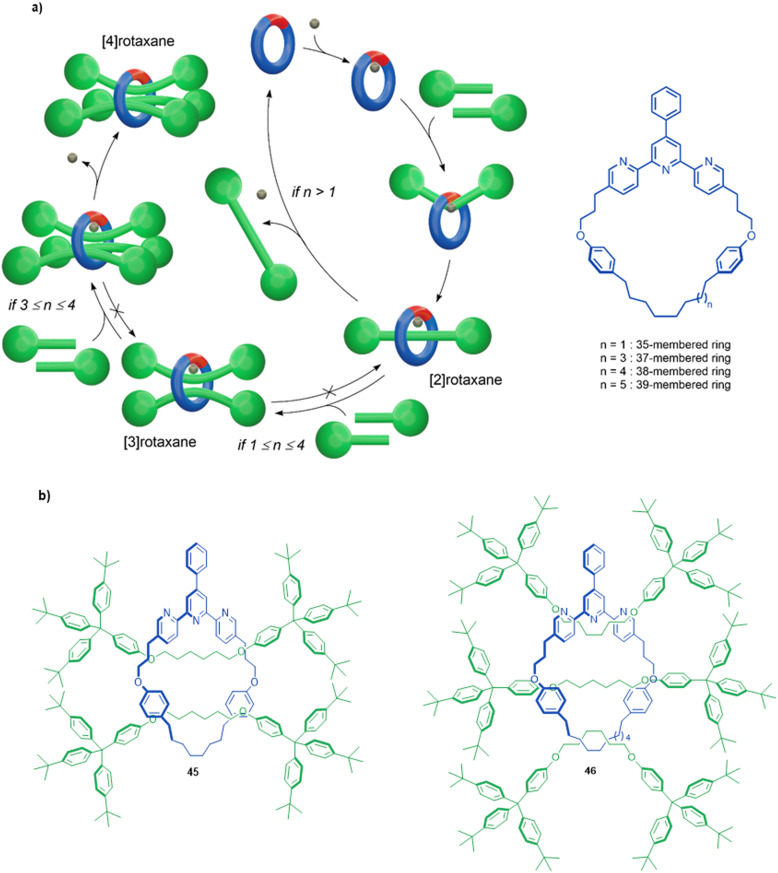
(a) Mechanism of the active template Ni(0)-mediated active template synthesis of rotaxanes with one, two or three axles threaded through a single macrocycle. (b) Doubly and triply threaded rotaxanes formed using this method.^102^

Active template synthesis by Ni-catalysed homocouplings of alkyl bromides can produce multiply threaded rotaxanes. Leigh and co-workers isolated doubly threaded rotaxane 45 in up to 51% yield using a terpyridine 35-membered macrocycle (Fig. 15a).^102^ During the process, [2]rotaxane 44 was also produced but the ratio of doubly- over singly-interlocked product could be increased by using 43 in large excess. The mechanism appears to proceed in a stepwise manner in which the [2]rotaxane is formed first, followed by binding of a second set of axle building blocks to the metal coordinated in the [2]rotaxane to yield [3]rotaxane 45 after a second reductive elimination.

A triply threaded [4]rotaxane 46 was obtained using the same strategy by increasing the size of the macrocycle from a 35-membered ring to either a 37- or a 38-membered ring (Fig. 15b).^103^ The increased size meant the macrocycle could accommodate the formation of a third axle within the cavity. [2]Rotaxane was not observed in the product distribution using the larger ring systems, only the doubly- and triply-threaded products. This suggests that the [2]rotaxane does not have stoppers large enough to prevent dethreading of the larger macrocycles unless the metal remains coordinated (which reduces the effective size of the cavity, and the coordinated metal can then promote the formation of the second and third axle; Fig. 15a). Also consistent with this, when a slightly larger 39-membered macrocycle was used only non-interlocked coupled products were observed. To date, rotaxane 46 is the sole example of a rotaxane containing more than two axles threaded through a single macrocycle.

**Fig. 16:**
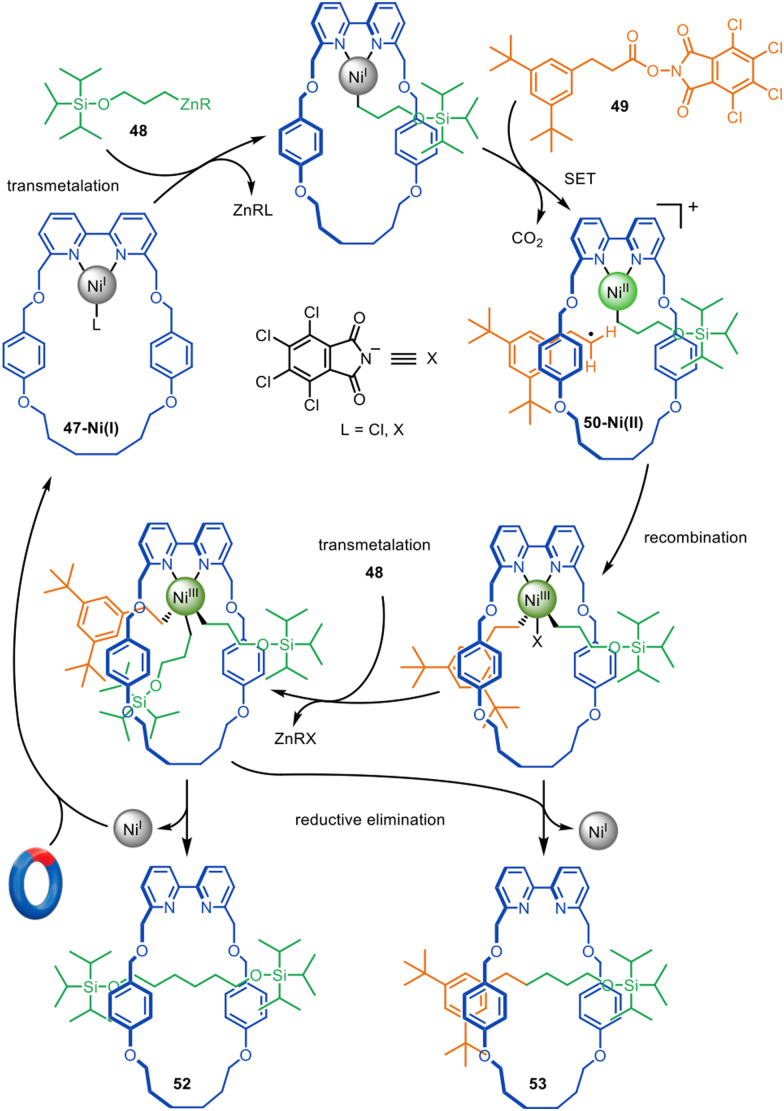
Mechanism of the active template synthesis of traceless [2]rotaxanes through the Ni(i)-mediated heterocoupling of alkylzinc and redox-active esters.^104^

The formation of traceless rotaxanes by active template synthesis was later extended by the Leigh group to the preparation of unsymmetrical axle [2]rotaxanes by the Ni-catalysed C(sp^3^)–C(sp^3^) cross-coupling of alkylzinc with redox-active esters (Fig. 16),^104^ a reaction introduced by Baran and co-workers.^105^ Transmetallation of Ni(i)-bipyridine macrocycle 47-Ni(i) with alkylzinc 48, followed by single electron transfer (SET) from the Ni(i) to the phthalimide group of redox-active ester 49 and consecutive decarboxylative fragmentation, leads to cationic intermediate 50-Ni(ii).^106^ Radical recombination renders Ni(iii) intermediate 51-Ni(iii), which undergoes reductive elimination through the macrocycle cavity to form [2]rotaxane 52, while regenerating the catalytic Ni(i) species. [2]Rotaxane 53 was also formed, resulting from the active template homocoupling of alkylzinc 48.

**Fig. 17:**
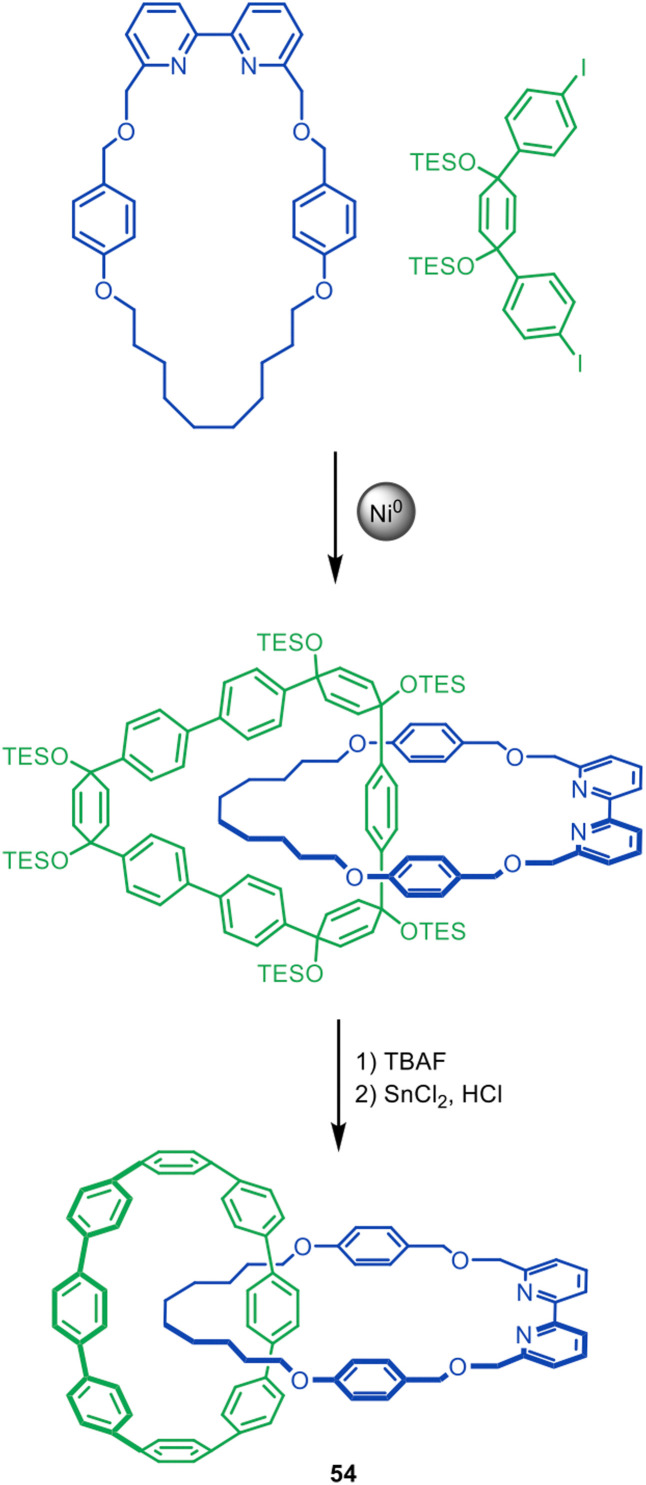
Kimizuka, Yagi, and Itami's Ni(0)-mediated active template synthesis of [8]cycloparaphenylene catenane 54.^107^

Recently, Kimizuka, Yagi and Itami reported a novel Ni-mediated homocoupling active template reaction employing a similar bipyridine macrocycle in the synthesis of catenane 54 (Fig. 17).^107^ The threading unit is based on Itami's previous synthesis of catenanes and a trefoil knot through a covalent scaffold approach,^108^ and features cyclohexa-1,4-diene units^109^ that are converted to phenyl groups to form the all-benzene ‘nanobelt’ of the final catenane.

### Zinc-, cobalt-, ruthenium- and rhodium-mediated active template synthesis

**Fig. 18:**
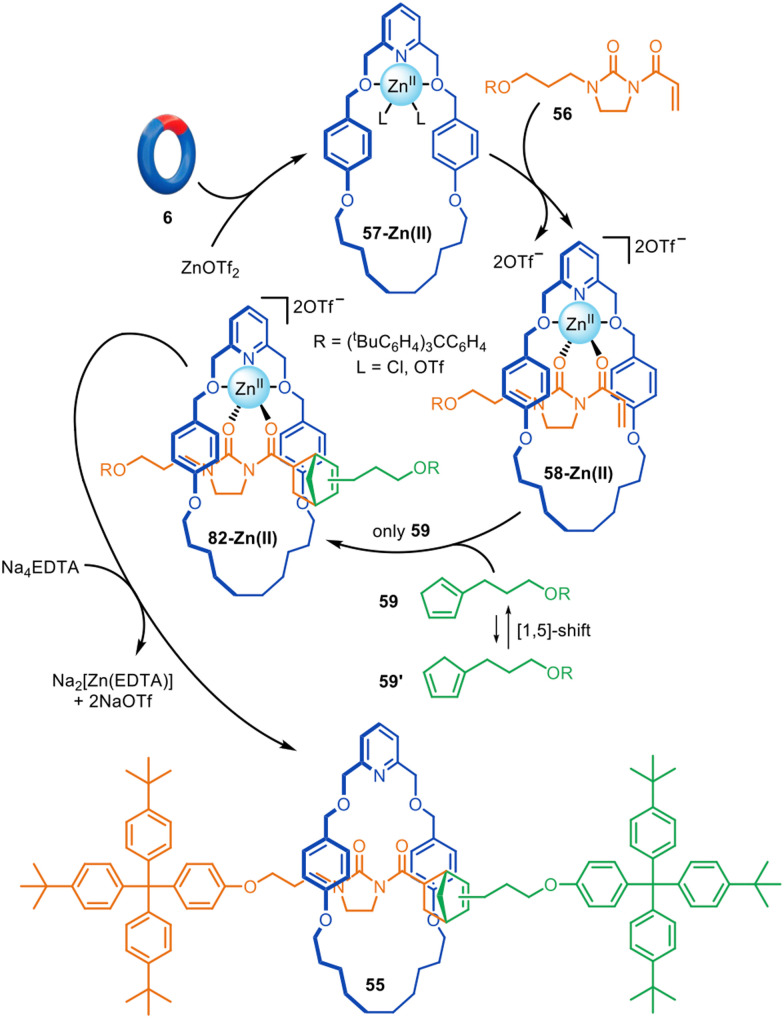
Active template synthesis of [2]rotaxanes through the Zn(ii)-mediated Diels–Alder of imidazolidones and cyclopentadiene derivatives.^110^

Although reactions based on copper, palladium and nickel catalysis are the most popular manifolds for active template synthesis, reactions promoted by other metals have also been explored. Zinc(ii) has been used for the active template synthesis of [2]rotaxanes by the Lewis acid-catalysed Diels–Alder cycloaddition of imidazolidones with cyclopentadiene derivatives.^110^ Rotaxane 55 forms in up to 91% yield using either Zn(ii) or Cu(ii) ions (Fig. 18). Upon coordination of dienophile 56 to macrocycle 57-Zn(ii), the activated threaded complex 58-Zn(ii) is formed. Cycloaddition with cyclopentadiene 59 produces 60-Zn(ii), with subsequent demetallation affording metal-free [2]rotaxane 55. Only one of the two interconverting isomers of 59 engages in the active template reaction with a 9 : 1 preference for the *endo* adduct observed.

**Fig. 19:**
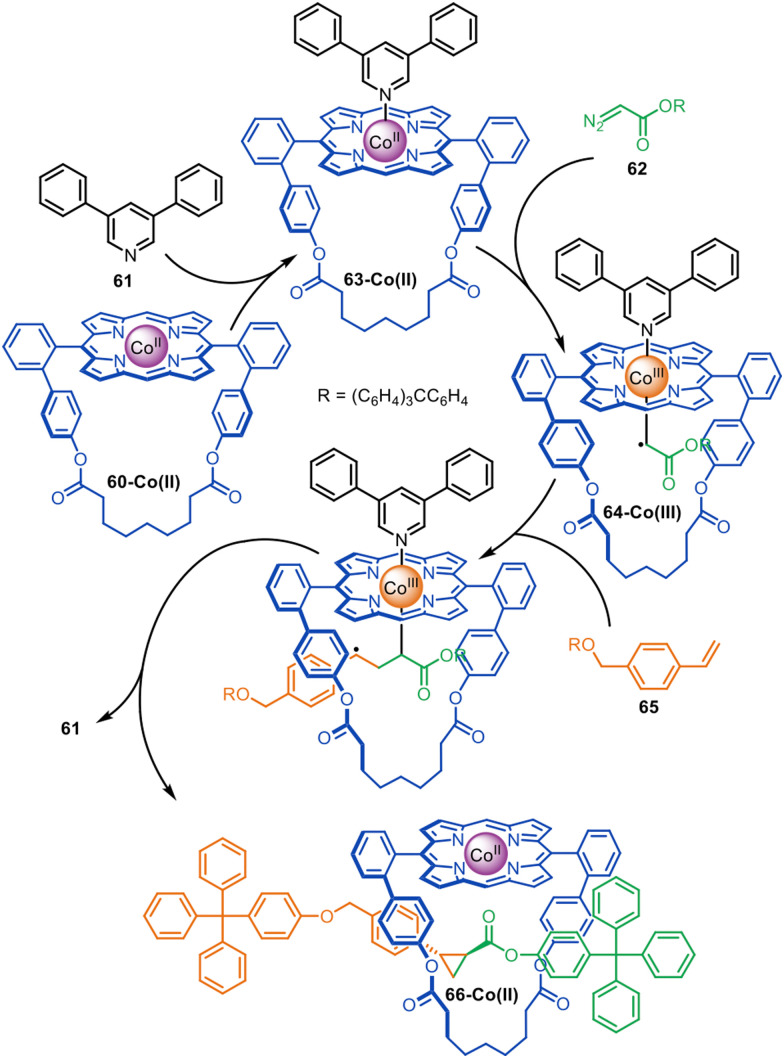
Megiatto's active template synthesis of [2]rotaxanes through the Co(ii)-mediated radical carbene transfer between diazo and styrene derivatives.^114^

A small number of active metal template reactions have been developed that proceed through radical mechanisms involving a redox process between the metal and the ligand. Co(ii)porphyrinates form nucleophilic radical carbenes that react with olefins to form cyclopropanes.^111–113^ Megiatto and co-workers exploited this chemistry for the active template synthesis of rotaxanes with macrocycle 60-Co(ii).^114^ The mechanism involves a radical carbene transfer reaction between diazo and styrene half-threads (Fig. 19). Coordination of 3,5-diphenylpyridine ligand 61 to the *exo*-face of the Co(ii)porphyrinate (it is too bulky to coordinate to the *endo*-face) causes diazo-acetate 62 to react with macrocycle 63-Co(ii) inside the cavity, leading to Co(iii)-carbene radical 64-Co(iii). Subsequent carbene transfer with styrene derivative 65 generates rotaxane 66-Co(ii). In the absence of 61 the yield of [2]rotaxane decreased from 95% to 74%,^115^ demonstrating the importance of coordination of an *exo*-ligand for promoting formation of the interlocked product over the free thread.

**Fig. 20:**
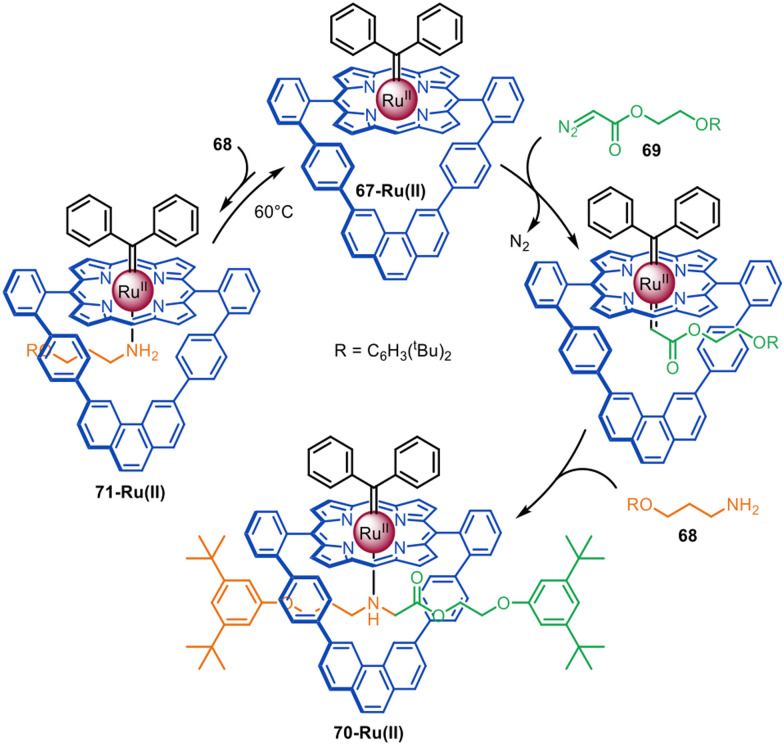
Megiatto's active template synthesis of [2]rotaxanes *via* Ru(ii)-mediated N–H carbene insertion.^116^

The same group reported that a Ru(ii)-porphyrinate macrocycle promotes N–H carbene insertion between amines and diazo half-threads in an active template fashion (Fig. 20).^116^ Ru(ii)-porphyrinate 67-Ru(ii) differs from 60-Co(ii) in that it is only composed of aromatic C–H bonds and therefore chemically inert to Ru(ii)-carbenoids. Introduction of a bulky carbene ligand *exo*- to the macrocycle forces reaction of diazo 69 to occur inside the cavity. Addition of amine 68 generates [2]rotaxane 70-Ru(ii) in quantitative yield.

**Fig. 21:**
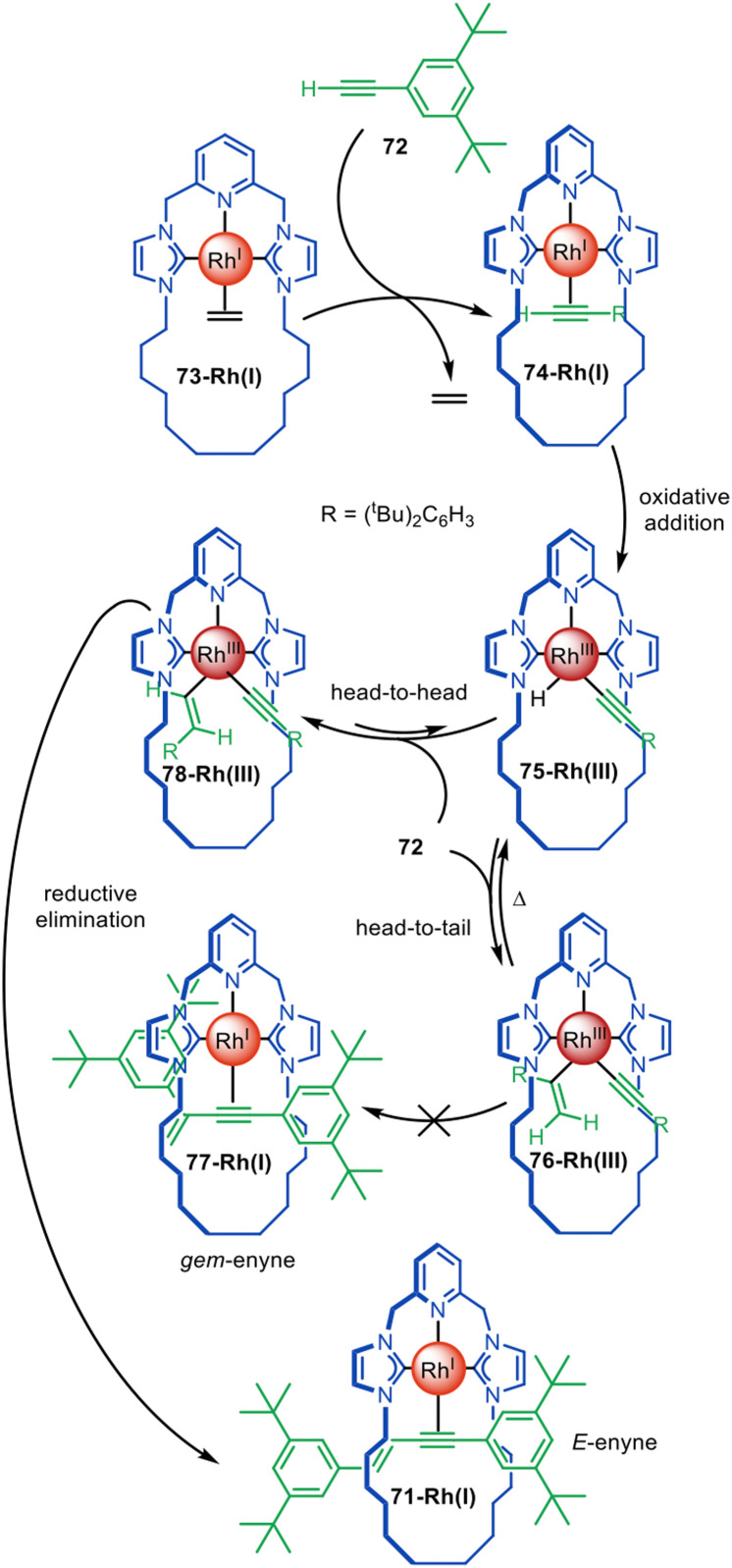
Chaplin's active template synthesis through the Rh(i)-mediated homocoupling of terminal alkynes to form a threaded *E*-enyne.^117,118^

The distinctive reactivity and catalytic properties of rhodium have been exploited for active template synthesis by the Chaplin group.^117–120^ They first described the formation of pseudorotaxane 71-Rh(i) by a Rh(i)-promoted terminal alkyne coupling reaction that forms an enyne axle (Fig. 21).^117^ Ligand exchange between terminal alkyne 72 and Rh(i) ethylene complex 73-Rh(i) leads to Rh(i)-alkyne complex 74-Rh(i), which undergoes oxidative addition to give 75-Rh(iii). A second equivalent of 72 inserts into the Rh–H bond in a ‘head-to-tail’ type mechanism to form a Rh(i) *gem*-alkenyl complex 76-Rh(iii) that is sufficiently stable to be isolated. However, upon heating to 80 °C, 76-Rh(iii) reductive eliminates and does not afford the expected Rh(i) *gem*-enyne pseudorotaxane 77-Rh(i), but instead quantitatively forms *E*-enyne pseudorotaxane 71-Rh(i). This suggests a mechanism in which β-hydride abstraction of 76-Rh(iii) occurs and the equilibrium is driven towards the formation of *E*-enyne Rh(iii) complex 78-Rh(iii), which can rapidly undergo reductive elimination to form 71-Rh(i). Only the *gem*-enyne product is formed when the reaction is performed with an acyclic CNC ligand. This study illustrates how active template synthesis can provide insights on ligand tuning to change reaction regioselectivity.^118^

**Fig. 22:**
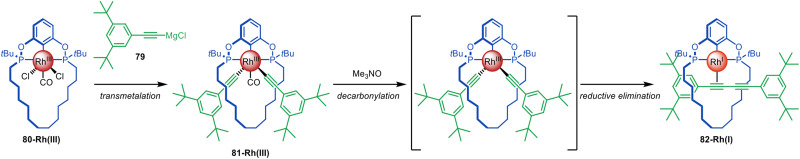
Chaplin's active template synthesis of diyne rotaxanes through Rh(iii)-mediated homocoupling of alkynyl Grignard reagents.^119^

The Chaplin group developed a Rh-mediated active template synthesis of diyne rotaxanes from alkynyl Grignard reagents (Fig. 22).^119^ Transmetallation of alkynyl Grignard 79 with a macrocyclic phosphinite pincer complex 80-Rh(iii) leads to 81-Rh(iii). Subsequent decarbonylation promoted by Me_3_NO affords diyne [2]rotaxane 82-Rh(i) through reductive elimination (81% yield over 2 steps). The group later reported a similar pincer complex with bulkier stoppers that could be converted to a hydrocarbon-based rotaxane.^120^ Demetallation followed by conversion of the phosphine ligands to thiophosphenes gave [2]rotaxanes, while a subsequent clipping reaction was used to synthesise a [2]catenane.

## Metal-free active template synthesis

A key feature of active template synthesis is that the reaction between the thread building blocks is substantially accelerated through a macrocycle's cavity compared to outside of it. This can also be achieved without metal catalysis. As long as the threaded product resulting from such a process is kinetically stable, it can provide a useful and effective means of spontaneously assembling rotaxanes under kinetic control. (Note: if the threaded structures are not kinetically stable under the reaction conditions, any rotaxane present is the result of conventional ‘passive template’ synthesis under thermodynamic control.)

### Mock's cucurbituril-promoted Huisgen azide–alkyne cycloaddition

**Fig. 23:**
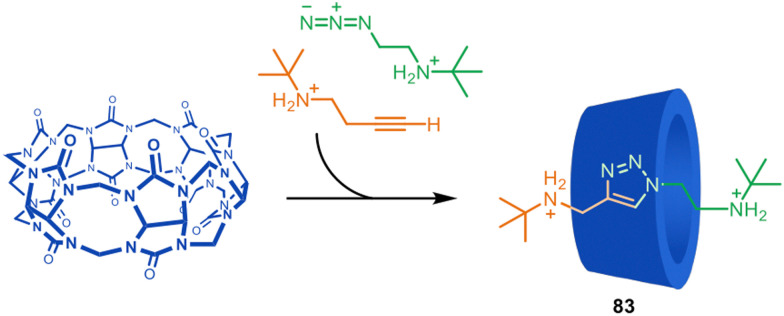
Mock's synthesis of a [2]rotaxane through cucurbituril-promoted Huisgen azide–alkyne cycloaddition of ammonium-functionalised axle building blocks.^121^

In 1989 Mock and co-workers carried out a rotaxane-forming reaction that can be considered a combination of passive ammonium ion template synthesis and metal-free active template synthesis.^121^ In a mechanistic investigation of the copper-free (Huisgen) azide–alkyne cycloaddition reaction of ammonium substrates catalysed by cucurbituril macrocycles, [2]rotaxane product 83 was formed by accelerated axle formation within the cucurbituril cavity (Fig. 23). The bulky substituents of the azide and alkyne reactants mean that the triazole axle cannot dethread. The cycloaddition was shown to be substantially accelerated (*ca.* 10^5^-fold rate increase) due to favourable orientation of the reactants within the cavity and strain-promoted reactivity.

**Fig. 24:**
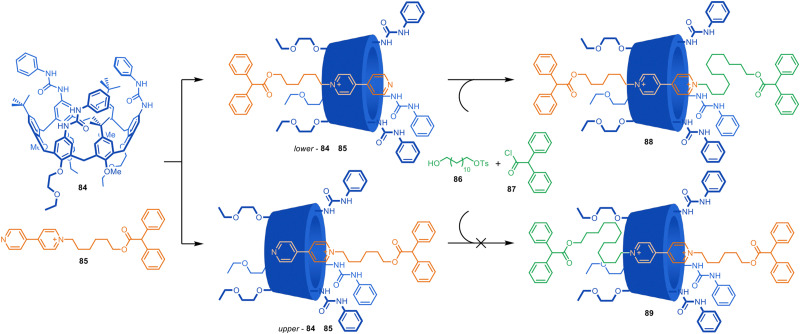
Credi and Arduini's cooperative capture synthesis of a [2]rotaxane.^124,125^

Similar complexation-driven effective molarity increases to accelerate axle-forming reactions have been used to assemble (poly)rotaxanes^122^ and for ‘cooperative capture’ synthesis.^123^ In addition to cucurbiturils, other 3D ‘molecular barrels’, such as cyclodextrins, pillararenes and calixarenes, have been used in such approaches. For example, Arduini, Credi and co-workers reported that a tri-urea-calix[6]arene accelerates the alkylation of a complexed pyridylpyridinium guest to form a rotaxane (Fig. 24).^124,125^ Macrocycle 84 forms a 1 : 1 complex with 85 in toluene, and upon addition of stoppering components 86 and 87, [2]rotaxane 88 is formed in up to 70% yield. The macrocycle accelerates alkylation of the pseudorotaxane 16× faster than the axle is alkylated in the absence of the macrocycle. The authors ascribe the rate enhancement to the increase of pyridine nucleophilicity inside the electron-rich cavity of 84 which contains multiple aromatic rings, as well as urea groups that could stabilize the transition state. No formation of 89 is observed due to the steric preferences of the non-symmetrical macrocycle. The authors later carried out the selective synthesis of all three possible orientational isomers of related calix[6]arene-based [3]rotaxanes.^126^

### Metal-free active synthesis of rotaxanes from crown ether-accelerated reactions of amines and nucleophiles

**Fig. 25:**
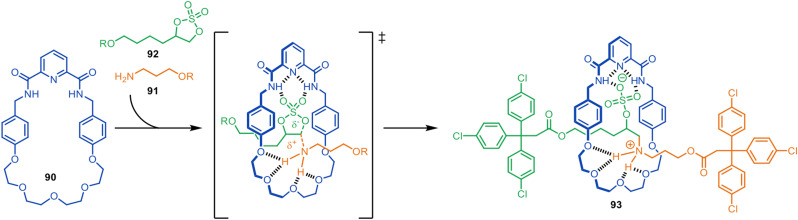
Metal-free active template synthesis of a [2]rotaxane through transition state stabilisation.^127^

In 2017, the Leigh group described the synthesis of [2]rotaxanes with a bifunctional macrocycle designed to stabilise the transition state of the axle-forming reaction (Fig. 25).^127^ The intention was that macrocycle 90, with a hydrogen bond donor pyridyl-2,6-dicarboxyamide unit at one end and an hydrogen bond acceptor oligo(ethylene glycol) chain at the other, would be able to promote the addition reaction of a primary amine 91 to a cyclic sulfate 92 through the cavity by stabilising the reaction transition state. The [2]rotaxane 93 was formed in 70% yield. Host–guest studies confirmed that the macrocycle does not strongly bind to either of the half-thread components, but rather stabilises the transition state of the reaction proceeding through the cavity.

**Fig. 26:**
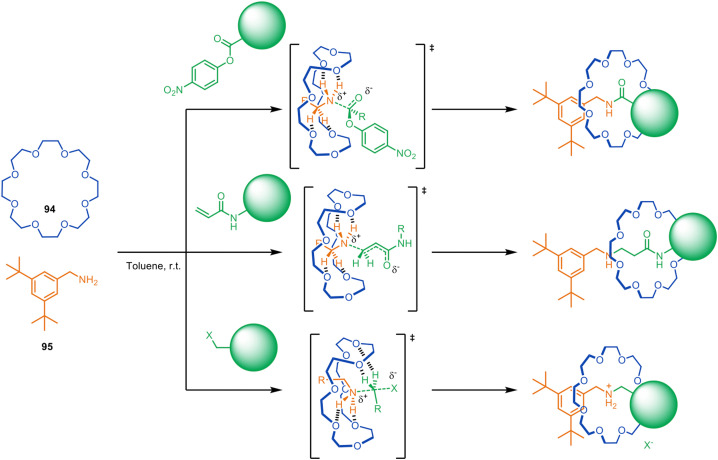
Metal-free active template synthesis of rotaxanes from primary amines, electrophiles and crown ethers.^128,129^

The control experiments used to interrogate the mechanism of formation of rotaxane 93 included the use of analogues of macrocycle 90 that were missing either the two amide groups (intended to stabilise the forming anion in the transition state) or the oligoethylene glycol chain (intended to stabilise the forming cation in the transition state). However, although replacing the glycol units with a hydrocarbon chain led to no rotaxane formation, a small amount (5%) of rotaxane was still formed if the macrocycle amide groups were changed to esters. This suggested that crown ethers might be able to promote the alkylation of a primary amine through the crown ether cavity. This is somewhat counterintuitive as amines bind only very weakly to crown ethers and, when protonated as ammonium groups (which do bind strongly to crown ethers) then there is no lone pair with which to act as a nucleophile. Nevertheless, this is exactly what happens (Fig. 26).^128^

Despite having modest binding constants for the thread building blocks, crown ethers such as 94 accelerate the reaction of primary alkyl- and benzylamines such as 95 with a series of electrophiles to form rotaxanes (Fig. 26).^128^ Kinetic studies show rotaxane formation with a benzylamine is ∼26× faster than the background thread-forming reaction. The process allows the reagent-less formation of crown ether rotaxanes in a matter of hours at room temperature, accomplished by simply mixing together the macrocycle and the two thread-forming building blocks in toluene.

The rotaxane formation is driven by crown ether hydrogen bonding stabilising the partial positive charges formed in the transition state, a process that has previously been observed in ethylene-glycol-oligomer-catalysed aminolysis reactions.^130,131^ The utility of the metal-free active template reaction was demonstrated by the one-step synthesis of (i) pH-switchable molecular shuttles *via* aza-Michael addition and (ii) crown ether-peptide rotaxanes *via N*-acylation (Fig. 26).^128^

The system was subsequently extended to other electrophiles, producing crown ether rotaxanes based on (thio)urea, carbamate, sulfonamide and phosphoramidate/phosphinamide-functionalised axles in up to 95% yield and >100 : 1 rotaxane-to-free-thread selectivity.^129,132^ It also proved possible to use crown ether catalysed *N*-arylation of primary amines with electron-deficient aryl halides to produce rotaxanes with aniline threads. This metal-free active template *N*-acylation strategy has been used for the single-step enantioselective synthesis of mechanically planar chiral [2]rotaxanes (see section on mechanical and topological chirality).^133^

**Fig. 27:**
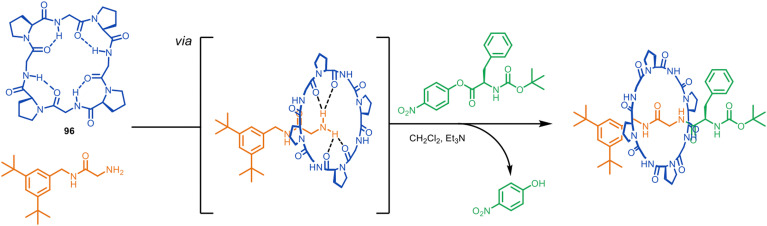
Numata's metal-free active template synthesis of wholly peptidic rotaxanes from a glycine nucleophile, a phenylalanine-nitrophenol ester electrophile and cyclo(proline) macrocycle.^134^

In an important recent development, the principle of enhancing the nucleophilicity of primary amines by hydrogen bonding to a macrocycle has been expanded from crown ethers to cyclic peptides.^134^ The Leigh group have previously threaded cyclo(proline) macrocycles such as 96 onto diammonium axles to form rotaxanes of cyclic peptides by passive template synthesis in up to 63% yield.^135^ Numata and coworkers found that this macrocycle-ammonium passive template motif could be adapted to metal-free macrocycle-amine active template synthesis in a similar manner to the crown ether-ammonium/amine system.^134^ With a glycine-derived amine nucleophile and phenylalanine-derived nitrophenol ester electrophile metal-free active template synthesis generated wholly peptidic rotaxanes (Fig. 27).^134^ The yields reported are very modest (∼1%), which the authors ascribe to the tendency of cyclic peptide oxygens to point out of the cavity and the presence of competing intermolecular hydrogen bonds, both of which limit the ability of the macrocycle to form strong hydrogen bonds with the amine. As well as being a strategy to mechanically interlocked peptides, the extension of metal-free active template synthesis from crown ethers to ammonium-binding cyclic peptides suggests that other ammonium-binding macrocycles (*e.g.* cucurbiturils, pillararenes, *etc*) may prove efficacious at accelerating the reaction of amines through a macrocyclic cavity for active template synthesis.

### Protein metal-free active template synthesis

**Fig. 28:**
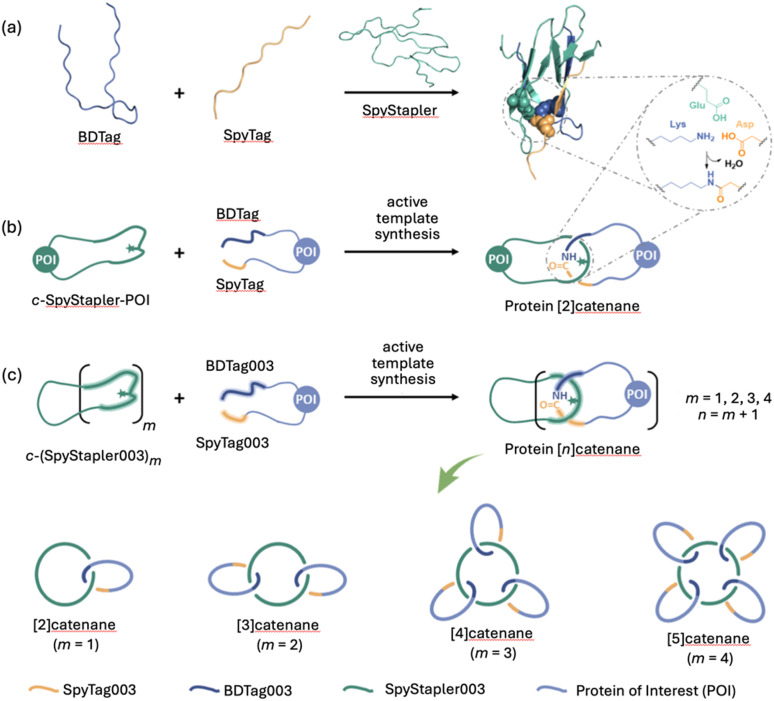
Zhang's metal-free active template synthesis of protein heterocatenanes.^136–138^ (a) SpyStapler-mediated isopeptide bond formation between SpyTag and BDTag.^137^ (b) Active template synthesis of a protein [2]catenane from a cyclic protein incorporating the SpyStapler sequence (*c*-SpyStapler-POI) and a linear protein terminated with the BDTag and SpyTag sequences at either end.^136^ (c) Higher order protein [*n*]catenanes (*n* = 2–5) assembled using a mutated SpyStapler sequence (SpyStapler003).^138^

The metal-free active template concept has also been applied to protein catenane synthesis.^136^ The Zhang group used protein engineering to design a catenane-forming reaction based on a SpyTag–SpyCatcher complex^137^ (Fig. 28a) where the catalytic unit embedded in a cyclic protein promotes isopeptide bond formation of a telechelic protein through its cavity (Fig. 28b). These entangled topologies displayed enhanced stability against proteolytic digestion, thermal and mechanical denaturing, without compromising their biological function. The group also demonstrated that introducing mutations in the catalytically active SpyStapler protein (SpyStapler003; Fig. 28c) led to improved efficacy of the active template reaction, which could be exploited for the synthesis of higher-order protein [*n*]catenanes in good yields (Fig. 28c).^138^

The group have also developed a novel protein (‘AT-Snoop’) for active template protein synthesis.^139^ AT-Snoop and AT-Spy both catalyse isopeptide bond-formation but react orthogonally using different proteins domains. The orthogonality was exploited for the synthesis of [*n*]heterocatenanes, including an asymmetric protein olympiadane.^138^

### Active template assembly of interlocked nanoscale toroids

**Fig. 29:**
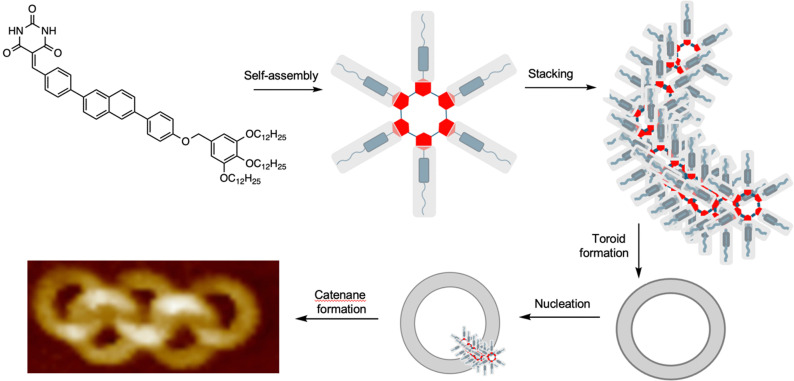
Yagai's self-assembling polycatenanes consisting of ∼13 nm diameter supramolecular rings.^140^ The molecules with a polar head group, rigid segment and non-polar tail assemble into rosettes that stack to form helical strands and toroids. The internal surface of the toroids seed the formation of new stacks, leading to supramolecular polycatenanes.^140^

A remarkable spontaneous assembly of nanoscale interlocked supramolecular toroids arises from a process closely related to metal-free active template synthesis (Fig. 29).^140^ Yagai and co-workers observed that molecules consisting of a barbiturate head group, a rigid central core and non-polar tail self-assemble into supramolecular hydrogen bonded rosettes. The rosettes stack in an off-set manner, leading to curvature that ultimately leads to the formation of toroids of ∼13 nm diameter. Nucleation of toroid assembly is quicker from sites within the toroid cavity than outside it (the fundamental principle behind active template synthesis; see Introduction and Fig. 2b) resulting in interlocking of the toroids. A range of interlocked materials with topologies were obtained, ranging from Hopf link [2]catenanes, to [5]ring olympiadanes and poly[*n*]catenanes.^140^

## Applications of active template synthesis

The special characteristics of active template synthesis include the lack of a requirement for persistent recognition motifs between the components in the interlocked product, and that the threaded structures need not be thermodynamically stable. This opens the way for the synthesis of interlocked molecules that are inaccessible through passive template synthesis. These features have led to active template synthesis being explored to make molecules for fields as diverse as molecular machinery, drug delivery systems, sensing devices, catalysis and materials science.

### Molecular shuttles

**Fig. 30:**
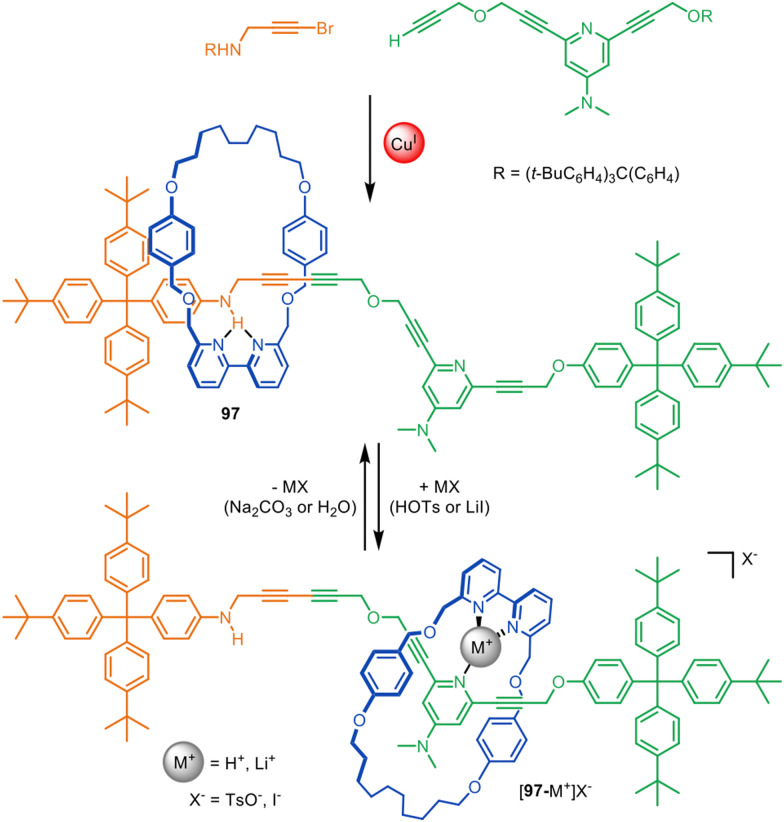
Active template Cadiot–Chodkiewicz synthesis of molecular shuttle 97. The position of the macrocycle on the axle is governed by relatively weak intercomponent interactions.^67^

Active template synthesis has been used to prepare a number of rotaxane-based molecular shuttles and switches; that is, rotaxanes in which the macrocycle incessantly moves from one well-defined site on the thread to another (resulting in a net change in ring position in the case of switches).^28,67,86,110^ The dynamic aspects of these systems have been reviewed elsewhere,^141,142^ but some advantages of using active template synthesis for their assembly are illustrated by switchable molecular shuttle 97 (Fig. 30).^67^

Molecular shuttle 97 was synthesised through an active template Cadiot–Chodkiewicz heterocoupling, with one alkyne unit containing an aniline and the other a dimethylaminopyridine (DMAP) group (Fig. 30).^67^ In its unprotonated form the macrocycle of 97 preferentially coordinates to the aniline site on the axle through a single hydrogen bond with an intercomponent binding energy of −3.9 kcal mol^−1^, which would be far too weak to thermodynamically drive rotaxane synthesis. Protonation or Li^+^ coordination of the DMAP group of 97 triggers the translocation of the macrocycle to the DMAP axle site. This illustrates the efficacy of active template synthesis for the preparation of rotaxane-based molecular shuttles with much weaker intercomponent interactions (and correspondingly faster dynamics) than those required for passive template methods.^143^

The active template Cadiot–Chodkiewicz reaction has been used to develop other mechanically interlocked structures without strong intercomponent binding interactions, such as the [*c*2]daisy chain rotaxane 27 described by Jasti and co-workers (Fig. 8). This structure can be switched from a comparatively stable contracted form at temperatures lower than −40 °C to an extended state at higher temperatures.^59^

Goldup and co-workers used active template CuAAC synthesis to prepare molecular shuttles in which the shuttling of a bipyridine ring between bidentate and tridentate coordinating sites^144^ could be controlled by metal ions with different preferential coordination geometries.^145^

Weiss, Guldi, Hayashi and co-workers have reported light-switchable molecular shuttling in a [2]rotaxane based on an electron donor–acceptor system composed of a Zn–porphyrin-based macrocycle and a fullerene-stoppered thread.^146^ An active template CuAAC reaction installs a triazole moiety in the axle that forms one binding site in the resulting shuttle.

Beer and co-workers have also reported porphyrin-based molecular shuttles synthesised by active template protocols. In those examples the position of a pyridine macrocycle (used to direct the CuAAC reaction) is controlled *via* the addition of a Lewis base or anion.^147^

### Mechanical and topological chirality

**Fig. 31:**
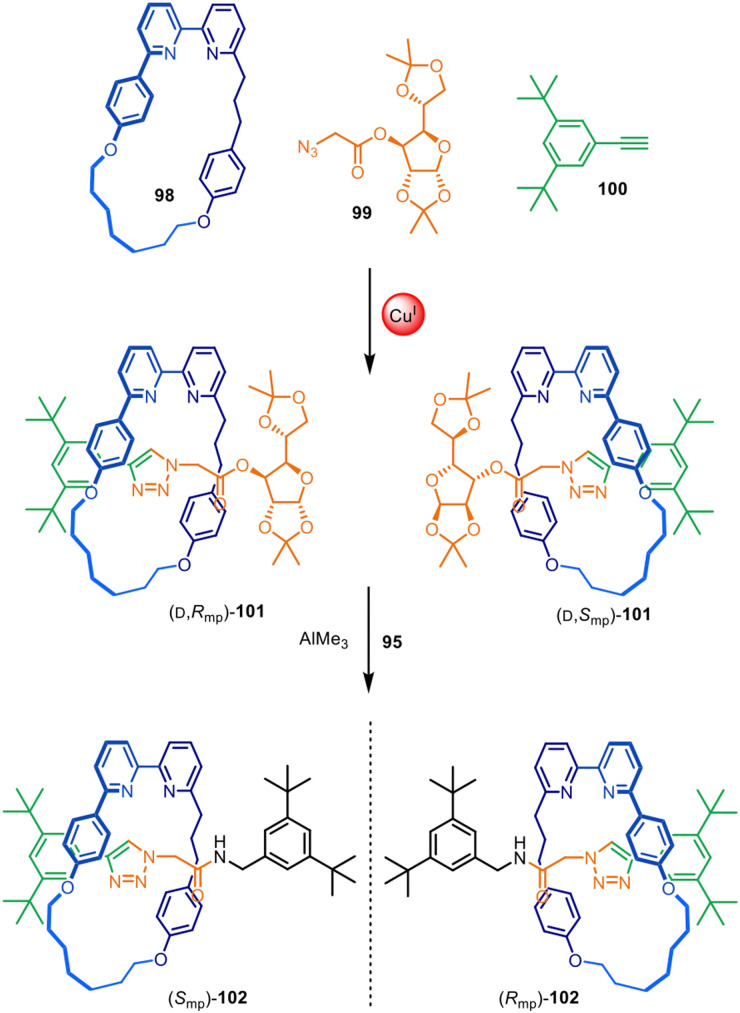
Goldup's active template synthesis of mechanically planar chiral rotaxanes (*S*_mp_)- and (*R*_mp_)-102.^148^

Active template synthesis has been used to make a range of mechanically and topologically chiral interlocked molecules, in which the chirality arises solely from the orientation of the interlocked components as opposed to point-chirality embedded in their structures. Active template synthesis is well-suited for this purpose as, like enantioselective synthesis, it is inherently under kinetic control. The Goldup group have made extensive use of active template CuAAC reactions to prepare mechanically planar chiral rotaxanes and catenanes (Fig. 31).^148^ A rotationally asymmetric bipyridine macrocycle (98), a sugar-based azide (99) and an alkyne (100) reacted in the presence of Cu(i) to produce a diastereomeric mixture of [2]rotaxane 101. Although the reaction did not impart stereoselectivity in the reaction outcome, it enabled simple separation of both diastereomers by chromatography. The chiral sugar could subsequently be replaced by an achiral amine nucleophile, removing the element of point chirality from the interlocked structures resulting in the isolation of both [2]rotaxane enantiomers (*S*_mp_)- and (*R*_mp_)-102.

**Fig. 32:**
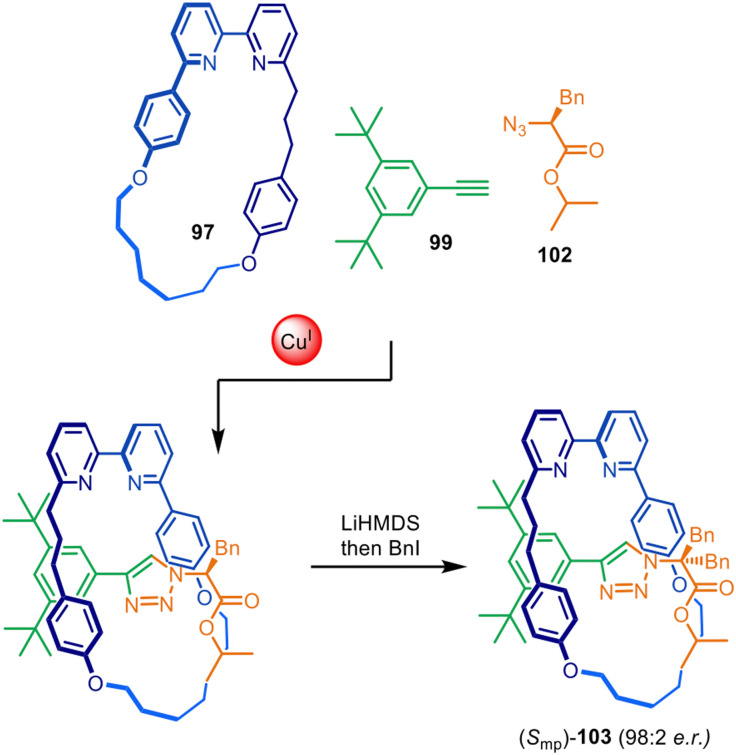
Goldup's diastereoselective active template synthesis of mechanically planar chiral rotaxane (*S*_mp_)-103.^149^

The approach was later expanded to diastereoselective synthesis of mechanically planar chiral rotaxanes by modifying the stereodirecting moiety in the half-thread building block (Fig. 32).^149,150^ Excellent diastereomeric ratios of 98 : 2 were obtained using chiral amino acid-derived azide 102. Post-assembly removal of the point chirality source by symmetrisation of the amino acid stereocentre rendered the enantioenriched mechanically planar chiral rotaxane (*S*_mp_)-103. Similar methodology was later applied to the stereoselective preparation of enantiopure topologically chiral catenanes.^151^

**Fig. 33:**
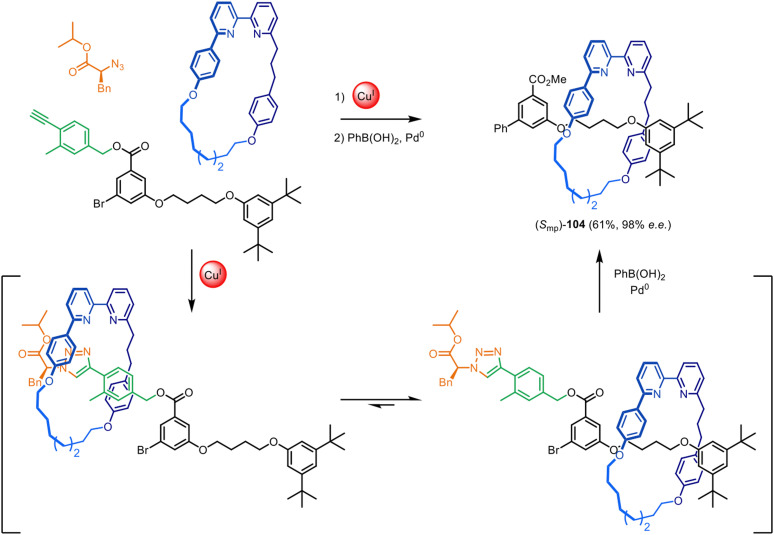
Goldup's mechanical interlocking chiral auxiliary for the active template synthesis of ‘impossible’ mechanically planar chiral rotaxanes.^25^

The Goldup group have also demonstrated that this chiral auxiliary can act as an mechanical interlocking auxiliary,^23–25^*i.e.* a group that facilitates the formation of the mechanical bond but is subsequently cleaved from the structure after shuttling of the macrocycle to a different part of the axle (Fig. 33).^25^ The auxiliary was removed to leave ‘impossible’ rotaxane^23,24^ (*S*_mp_)-104, with no recognition motif between the axle and macrocycle. The group also investigated attaching the chiral auxiliary to the macrocycle, rather than an axle-forming component.^152^ This could be used to direct the formation of both mechanically planar chiral rotaxanes and topologically chiral catenanes with good stereoselectivity (typically >90% ee).

CuAAC active template synthesis has also been used by the Papot group for the diastereoselective synthesis of [1]rotaxanes (‘molecular lassos’).^153,154^ The point-chiral stereogenic unit has not yet been removed, meaning that a mechanically planar chiral [1]rotaxane with chirality solely arising from the mechanical bond remains to be achieved.

The active template mechanical interlocking chiral auxiliary systems developed by the Goldup group have been used to make a number of catenanes and rotaxanes with other, previously unexplored, types of chirality, including co-conformationally ‘pseudo-topologically’ chiral catenanes,^155^ mechanically (but not topologically) planar catenanes,^156^ mechanically axially chiral catenanes and noncanonical mechanically axially chiral rotaxanes.^157–159^ The topological aspects of these fascinating systems has recently been reviewed elsewhere.^160^ The versatility and efficiency of the active template approach has facilitated the exploration of new types of chiral interlocked molecules, leading to a better understanding of aspects of molecular asymmetry.

**Fig. 34:**
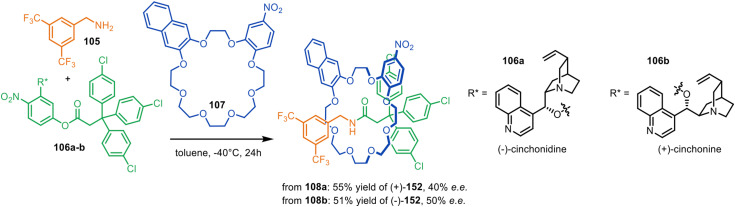
Single-step asymmetric metal-free active template synthesis of mechanically planar [2]rotaxanes.^161^

The Leigh group reported a single-step asymmetric synthesis of mechanically planar chiral rotaxanes by using primary amine 105, activated ester 106a–b with a chiral leaving group and achiral crown ether macrocycle 107 with *C*_s_ symmetry in an application of metal-free active template synthesis (Fig. 34).^161^ The incorporation of cinchonidine pseudoenantiomers as directing groups enabled the synthesis of either enantiomer of [2]rotaxane 108 in 40–50% ee. X-Ray crystal structures and computational modelling indicated π-stacking between the naphthalene ring of the macrocycle and the electron-deficient aromatic stopper helps to direct the reaction stereoselectivity.

A different approach to mechanically planar chiral rotaxanes, also exploiting metal-free active template synthesis, was developed by Tian and Zhu.^162^ Modified bis(chloroaryl) crown ethers were used as catalysts for the spontaneous formation of [2]rotaxanes using a range of electrophiles and amine nucleophiles. The prochiral interlocked molecule (with an unsymmetrical axle but a rotationally symmetric ring) then underwent catalytic desymmetrisation through a Pd-catalysed asymmetric Suzuki–Miyaura reaction to yield the chiral rotaxanes in up to 97 : 3 er.

### Asymmetric, diastereoselective and other types of catalysts

**Fig. 35:**
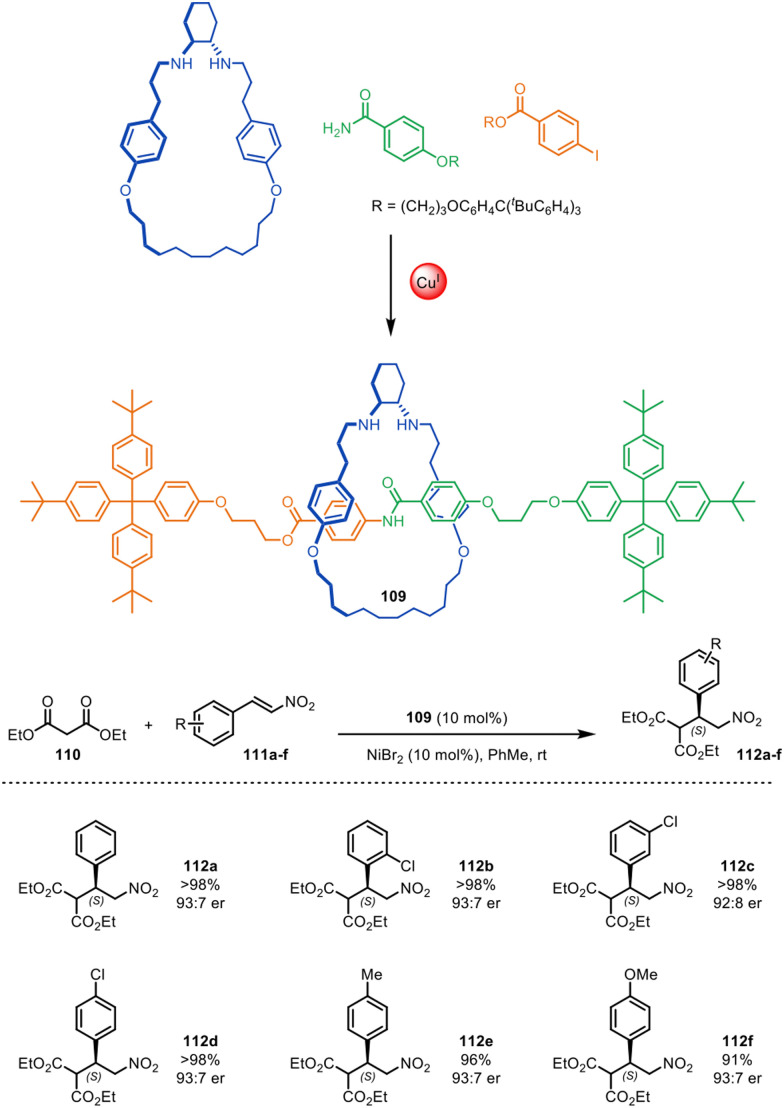
Mechanically chiral [2]rotaxane 109, synthesised through a Goldberg active template reaction, and its scope as a ligand for the nickel-catalysed enantioselective Michael addition of diethyl malonate and trans-β-nitrostyrenes.^73^

In 2015, Leigh and co-workers reported the first use of a mechanically chiral [2]rotaxane ligand for enantioselective catalysis, synthesised *via* active template methodology.^73^ [2]Rotaxane 109 (Fig. 35) was prepared through Cu(i)-mediated Goldberg active template synthesis. The chiral interlocked structure has an endotopic binding site on the macrocycle that can bind various transition metals. The chiral rotaxane ligand was used for the Ni(ii)-catalysed enantioselective Michael addition of diethyl malonate 110 to various nitrostyrene derivatives 111a–f, affording products 112a–f in good yield and enantiomeric enrichment (typically >98% yield and 93 : 7 er). Chiral induction with the rotaxane ligand was significantly more effective than with a comparative acyclic chiral ligand (68 : 32 er), although considerably longer reaction times were needed with the rotaxane (27 days compared to 2 days). Both the strong enantioselectivity and the slow reaction times can be attributed to the sterically hindered binding pocket of the rotaxane which results in a catalytic site where the chirality is well-expressed in all three dimensions.

**Fig. 36:**
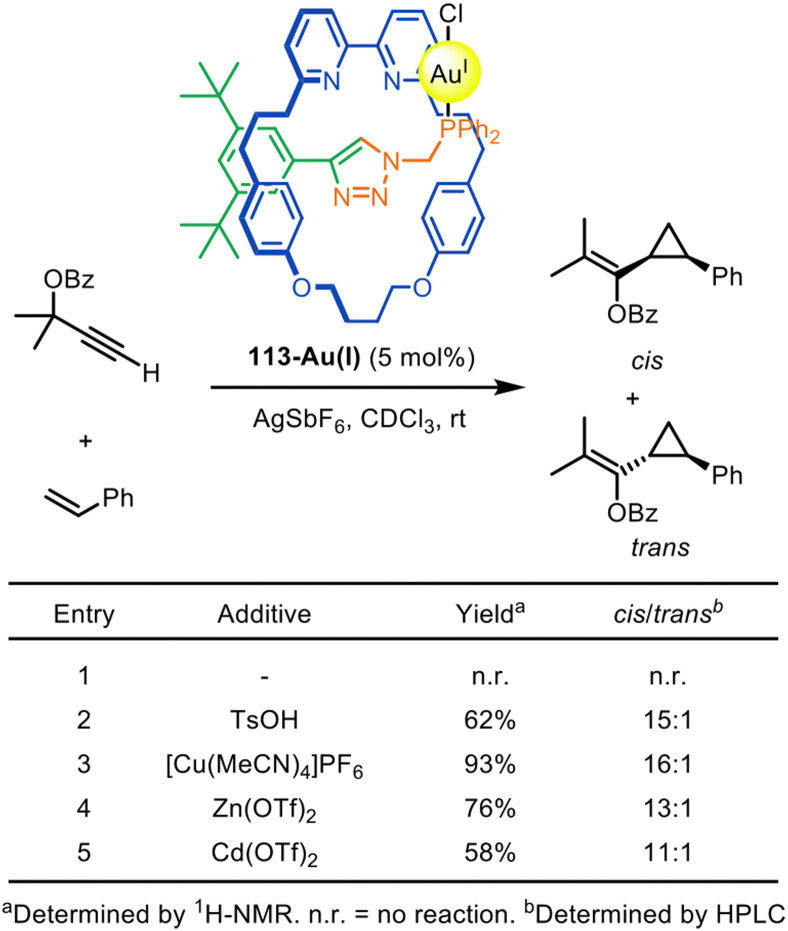
Goldup's [2]rotaxane precatalyst 113-Au(i) prepared by an active template CuAAC reaction, and the effect of additives on its catalytic efficacy in the Toste–Ohe–Uemura cyclopropanation reaction.^163^

The Goldup group have demonstrated that chiral rotaxanes can be used as ligands for diastereoselective catalysis. An active template CuAAC reaction was used to synthesise the interlocked ligand for [2]rotaxane-gold catalyst 113-Au(i).^163^ The catalytic activity could be switched on by the addition of catalytically innocent metal cations (Fig. 36). 113-Au(i) was shown to catalyse Toste's Au(i)-mediated modification of the Ohe–Uemura cyclopropanation reaction.^164,165^

**Fig. 37:**
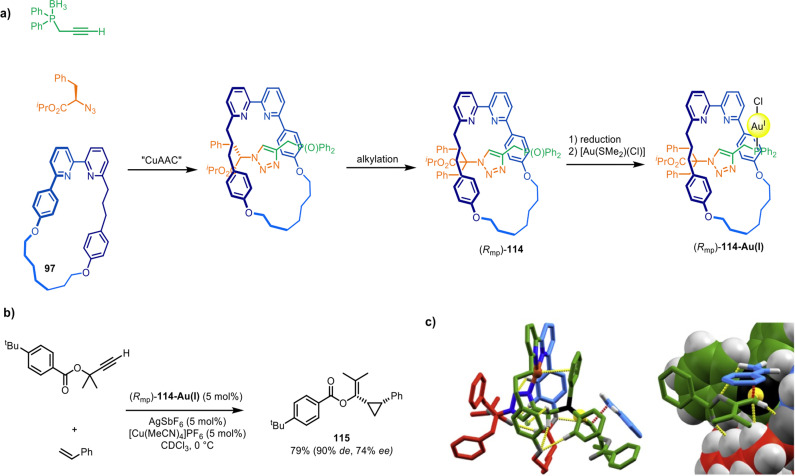
(a) Goldup's active template synthesis of mechanically planar chiral rotaxane precatalyst (*R*_mp_)-114-Au(i). (b) Example of a diastereoselective and enantioselective Toste–Ohe–Uemura cyclopropanation reaction promoted by 114-Au(i). (c) Computationally modelled transition state.^166^

Building on this system, Goldup and co-workers described a catalyst for asymmetric synthesis that relies on the mechanically planar chirality of the rotaxane ligand as the sole source of chiral induction.^166^ Ligand (*R*_mp_)-114 was synthesised *via* a diastereoselective active template CuAAC reaction^149^ (Fig. 37a) (see section on mechanical and topological chirality). Upon coordination to Au(i) and activation with a cofactor, (*R*_mp_)-114-Au(i) proved an effective catalyst for the Au(i)-mediated Toste–Ohe–Uemura cyclopropanation, generating a range of cyclopropanes (*e.g.*115) in good yields and stereoselectivities (62–92% de for the *cis* diastereomer, 9–77% ee; Fig. 37b). Modelling and transition state calculations suggest that (*R*_mp_)-114-Au(i) possesses a well-defined and rigid chiral environment around the metal centre (Fig. 37c).

**Fig. 38:**
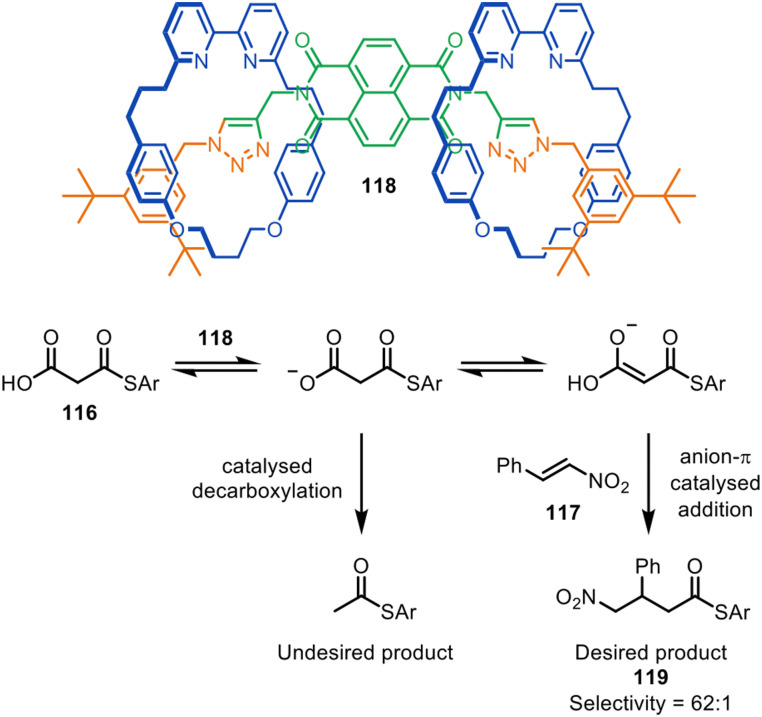
Goldup's [3]rotaxane anion–π organocatalyst 118 for the Michael addition of malonic acid monothioester to β-nitrostyrene.^167^

The Goldup group have also reported [2]- and [3]rotaxane organocatalysts, synthesised *via* CuAAC active template reactions, that promote the anion–π catalysed Michael addition of malonic acid monothioester 116 to β-nitrostyrene 117 (Fig. 38). The selectivity of 62 : 1 with [3]rotaxane catalyst 118 for addition/decarboxylation product 119 was comparable with the most effective non-interlocked catalysts previously reported.^167^ Computational studies suggested that the high selectivity observed may be due to π-stacking between the protonated bipyridine group and the naphthalene diimide.

In catalysis applications other than stereoselective catalysis, Schmittel and co-workers have exploited the triazole units formed during CuAAC active template reactions to create binding sites for an allosterically regulated silver(i) catalyst.^168^ Catalysis by the [2]rotaxane was regulated by steric restrictions on the conformational mobility of the components inhibiting the formation of catalytically active species. They have also described the design of a three-input logic AND gate with catalytic output using a [2]rotaxane shuttle prepared by active template synthesis.^169^

### Sensing and molecular recognition

**Fig. 39:**
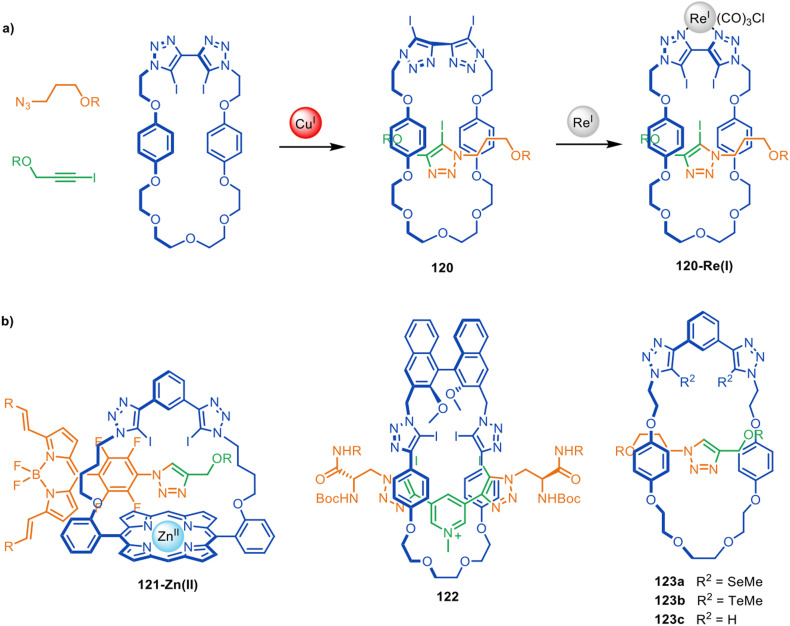
(a) Beer's active template synthesis of [2]rotaxane 120-Re(i) for anion sensing. (b) Other examples from the Beer group of halogen- and chalcogen-bonding rotaxane hosts prepared by active template synthesis.^170,179,180,182^

Beer and co-workers have used active template synthesis to assemble [2]rotaxane hosts for molecular recognition and sensing.^170^ Based on their earlier halogen bonding host molecules,^171,172^ halogen bond donating units were incorporated in both the macrocycle and axle components of a rotaxane to generate a 3D anion binding pocket. Modification of the active template CuAAC reaction installed a iodotriazole ring as part of the rotaxane axle^173^ with a bis-iodotriazole macrocycle, generating [2]rotaxane 120 in 25% yield (Fig. 39a). Exotopic coordination of a Re(i) atom to the triazole ligands was then used to orient the iodine atoms towards the inner cavity of the macrocycle. Host 120-Re(i) preferentially bound size- and shape-complementary Cl^−^ anions over other halide and acetate anions. The group have subsequently explored structurally related rotaxanes that bind halides in the presence of water,^174,175^ hydrogen and halogen bonding rotaxanes for alkali metal-halide ion-pair sensing,^176,177^ and a ferrocene-functionalised rotaxane for electrochemical sensing of bromide.^178^ Rotaxanes 121-Zn(ii) and 122 (Fig. 39b) proved to be effective fluorescent sensors^179^ and chiral anion receptors,^180^ respectively, and several related rotaxanes have been prepared that can switch between cationic and anionic binding modes.^181^

The Beer group have also demonstrated that Cu(i) binding to alkyl-selenium or alkyl-tellurium moieties in a macrocycle can be used to direct CuAAC active template synthesis through the macrocycle to form chalcogen-derivatised rotaxanes such as 123a–b (Fig. 39b).^182^ The resulting rotaxanes bind hard and soft anions in a broad range of environments.

A range of hydrogen bonding motifs unrelated to the active template assembly process have been incorporated into mechanically interlocked hosts for the recognition of small molecules and anions. McClenaghan, Tucker and co-workers prepared [2]rotaxane systems containing barbital recognition sites, such as 17 (Fig. 6b), *via* active template copper-catalysed CuAAC and Glaser reactions.^47,183^ Beer *et al.* constructed a neutral [2]rotaxane host for anionic guests using a hydrogen bond donating indolocarbazole unit as part of the axle.^184^ A [2]rotaxane host including an amide binding site was used by the Ghosh group for the electrochemical sensing of anions.^185^ Jasti and co-workers used a Cu-catalysed active template reaction for a family of [2]rotaxanes in which fluoride-^70^ or thiolate-^186^ induced dethreading results in the switching on of fluorescence.

The Goldup group have used CuAAC active template synthesis to build a number of rotaxanes for anion^187^ and cation^188–190^ binding. Some of the interlocked ligands stabilise distorted metal coordination geometries with unusual coordination numbers due to the sterically restricted 3D shape of the binding pocket.^189^ This is exploited in a Pt(ii)-rotaxane complex, in which the metal is stabilised towards oxidation and acts as a reversible stimuli-responsive phosphorescent sensor for Ag(i) ions.^190^

### Prototypes of drug delivery systems

**Fig. 40:**
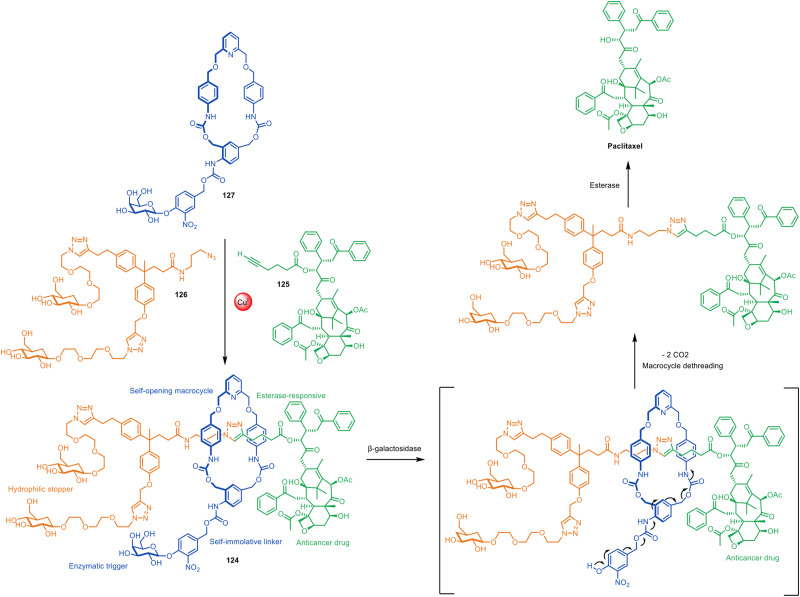
Papot and Leigh's active template CuAAC synthesis of β-galactosidase-cleavable [2]rotaxane 124, which releases paclitaxel in tumour cells.^191^

The lack of need for specific functional groups on the axle of rotaxanes using active template synthesis led to the design of enzyme-sensitive [2]rotaxane 124 by Papot, Leigh and co-workers for the controlled release of the anticancer drug paclitaxel in tumour cells (Fig. 40).^191^ Prodrug stopper 125 and hydrophilic fragment 126 were coupled through a self-immolative macrocycle (127) containing a Cu(i)-coordinating pyridine group. While the non-interlocked thread undergoes rapid esterase hydrolysis in plasma, the macrocycle in 124 acts as a shield, protecting the ester group in the axle from enzymatic degradation. However, cancer cells overexpress β-galactosidase, which triggers a cascade of reactions that culminate in the opening of the macrocycle and release of the active form of the drug.

Besides providing temporary shielding of an axle, rotaxane formation can be used to transiently modify the solubility and absorption properties of molecular components. Lewis *et al.* reported the use of an a CuAAAC active template [2]rotaxane as a triggerable cage for G-quadruplex (G4) DNA binders.^192^ The [2]rotaxane has better cell permeability than the analogous non-interlocked DNA binder, but lower cytotoxicity (probably due to the macrocycle inhibiting access to the G4 DNA binder). Enzymatic or light-triggered cleavage of one of the rotaxane stoppers liberates the G4 DNA binder within the cell, restoring its binding properties and cytotoxicity with high spatiotemporal control. Goldup, Tavassoli and coworkers have designed a triggered Curtius rearrangement immolative system where macrocycles can be functionalised at a late stage to incorporate a range of different cargos and trigger units.^193^ The approach was demonstrated with bipyridine macrocycles for CuAAC active template reactions and, notably, with crown ethers for metal-free active template synthesis.

### Sheathed ‘molecular wires’: rotaxanes and catenanes with extended-conjugated components

Active template alkyne homocoupling and alkyne–alkyne heterocoupling reactions have been used to prepare a wide range of polyyne rotaxanes with π-conjugated axles. The groups of Anderson, Tykwinski,^51,61,62,71^ and Gladysz^52,58,59^ have explored the synthesis of polyyne rotaxanes using active template Glaser and Cadiot–Chodkiewicz couplings. Long polyyne chains are prone to degradation and a threaded sheath structure can provide substantially improved stability.^61–63^ Sheathed polyynes also show some modifications in photophysical behaviour compared to the non-interlocked components,^64,68^ leading to their investigation as molecular wires.

**Fig. 41:**
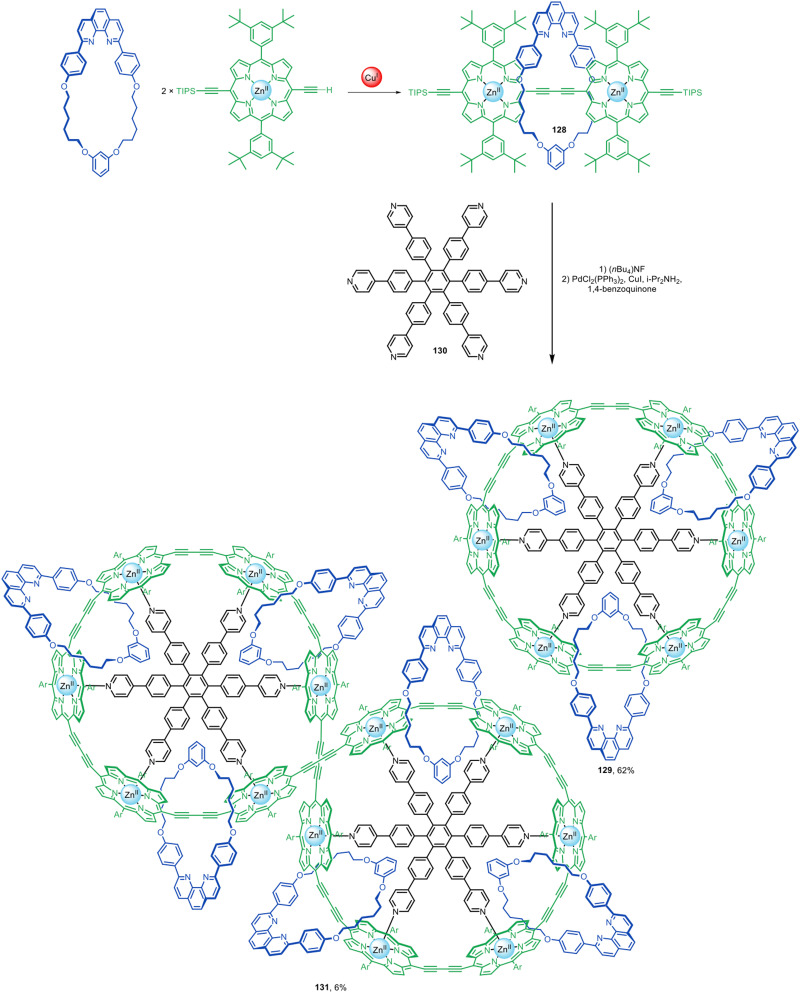
Anderson's assembly of [4]- and [7]catenanes from [2]rotaxanes prepared by active template Glaser homocouplings.^60^

The Anderson group have used active template Glaser couplings for the synthesis of π-conjugated rotaxanes with Zn(ii)-porphyrin stoppers at either end (Fig. 41).^60^ Porphyrin [2]rotaxane 128 was elaborated into [4]catenane 129 in 62% yield using 130 as a radial template for the interlocked architecture. [7]Catenane 131 was also formed as a Vernier template by-product of the synthesis.

**Fig. 42:**
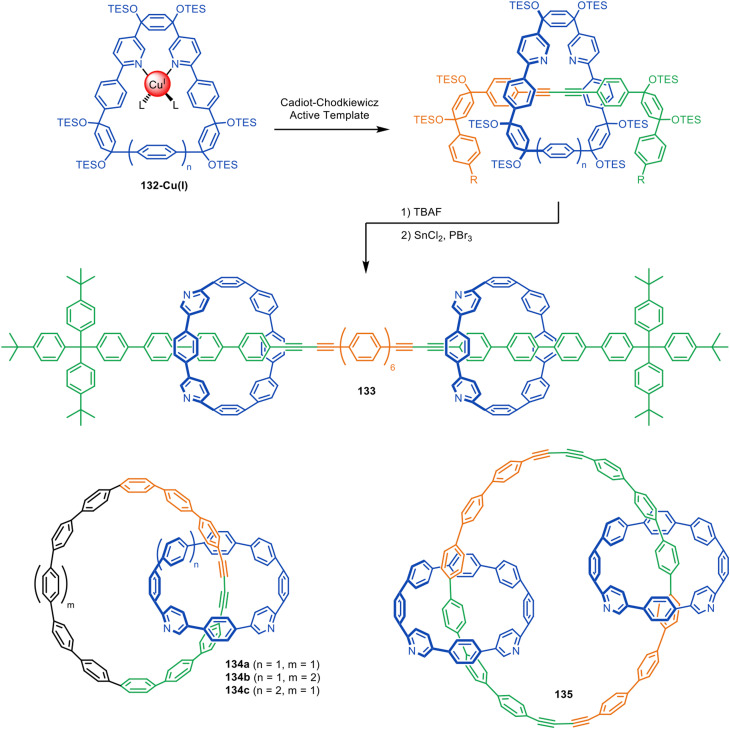
Jasti's assembly of interlocked nanocarbons *via* active template Cadiot–Chodkiewicz synthesis.^194^

The Anderson and Jasti groups have both used masked phenyl and alkyne units to extend the number of π-conjugated units in the axle and increase the diversity of accessible polyyne rotaxane and catenane structures. Jasti and co-workers used cyclic precursors to [*n*]cycloparaphenylene nanohoops for the synthesis of π-conjugated rotaxanes and catenanes *via* active template Cadiot–Chodkiewicz heterocouplings (Fig. 42).^194^ The bent cyclohexadiene moieties in 132 facilitate endotopic metal binding, which is difficult to achieve with cylindrical [*n*]cycloparaphenylenes. Reductive aromatisation of the cyclohexadienes furnishes the nanohoops in [3]rotaxane 133 and catenanes 134–135. The group later expanded the methodology to CuAAC active template synthesis, producing triazole catenanes in high yields.^195^

**Fig. 43:**
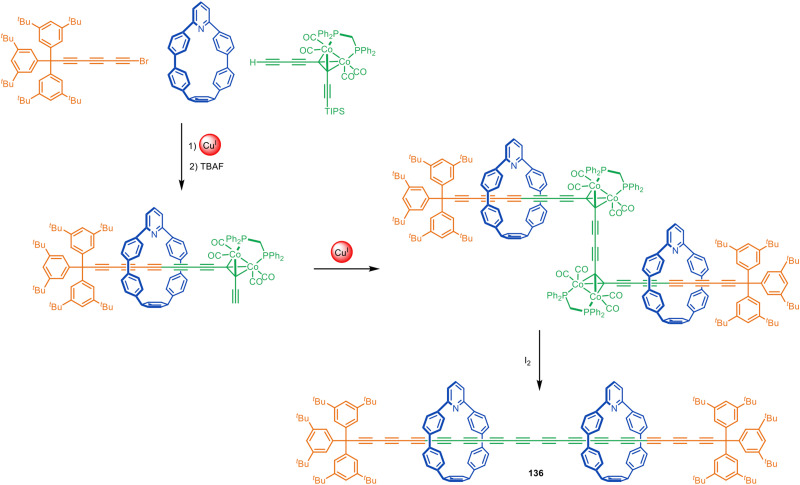
Anderson's extended polyyne rotaxane 136 synthesised *via* a Cadiot–Chodkiewicz active template reaction followed by oxidative unmasking of the bridging alkynes.^198^

Anderson and co-workers have employed indane^196^ and bicyclo[4.3.1]decatriene^197^ as photolabile masked-alkyne equivalents as well as dicobalt carbonyl complexes that can be unmasked by oxidative decomplexation.^198^ These synthons have been used in active template Glaser and Cadiot–Chodkiewicz syntheses of rotaxanes and catenanes, including polyyne [3]rotaxane 136 (Fig. 43) which has 14 contiguous alkyne units in the axle stabilised by the nanohoop sheath. The strategy was later used to prepare a polyrotaxane with an extraordinary 34(!) contiguous triple bonds in the axle and four threaded macrocycles.^199^ This polyyne rotaxane is in a length regime where the electronic properties of polyynes converge to those of carbyne. The rotaxane displayed electronic properties similar to those predicted for an infinite carbyne, illustrating the potential of polyyne rotaxanes to act as molecular wires.

The synthesis of catenanes with this method has also been reported, however, unmasking of the alkynes resulted in a complex mixture of products that could not be purified.

The CuAAC active template reaction has been used by the groups of Ngo, D’Souza and Goldup for the synthesis of porphyrinoid rotaxanes.^200,201^ These compounds displayed similar photophysical properties to the free thread component, but the threaded macrocycles inhibit intermolecular interactions between axles including π-stacking-driven aggregation and ligand-driven dimerisation.

### Modification of chemical and physical properties

Mechanically interlocking molecular components can significantly alter both chemical and physical properties.^202^ Active template synthesis is particularly useful in this regard as it enables components with otherwise weak binding affinities to be locked in close proximity, a situation that is hard to achieve in other ways.

**Fig. 44:**
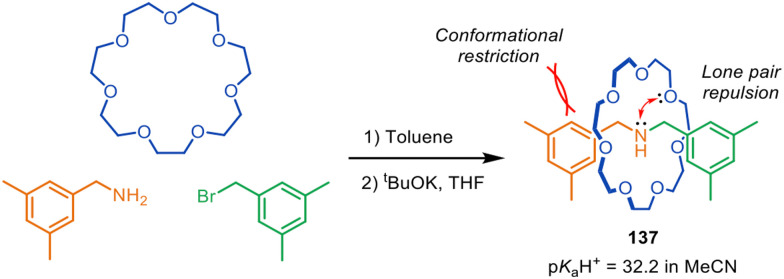
Spontaneous assembly by metal-free active template synthesis of compact rotaxane superbase 137.^203^

Leigh and co-workers have used metal-free active template synthesis to make compact amine-crown ether rotaxanes that exhibit exceptional basicity (Fig. 44).^203^ The super-basicity results from three main factors: (1) the formation of strong hydrogen bonding and electrostatic interactions in the protonated salt of the rotaxane; (2) the forced proximity (and lack of conformational and co-conformational freedom) of the amine and ethylene glycol functional groups, resulting in electrostatic repulsion between the interlocked macrocycle and thread; (3) a high degree of preorganisation of the components in the free-base rotaxane that favours amine protonation.

A range of compact rotaxane superbases was prepared by metal-free active template in 1–3 steps from commercially available starting materials. The compact rotaxane superbases had p*K*_a_H^+^ values as high as 32.2 for 137 in acetonitrile (13 p*K*_a_H^+^ units higher than the non-interlocked components, substantially larger than that of typical crown ether-amine rotaxanes), which are comparable to some phosphazene superbases. The highest basicities were generated with the smallest macrocycle (21-crown-7) and the most electron-rich dibenzylamine axles. The most efficacious compact rotaxane superbases showed minimal nucleophilicity and excellent hydrolytic stability in comparison to conventional commercial superbases.

Rotaxane formation, either by active or passive synthesis, can also be used to tune photophysical properties. An illustration is the Goldup and Zysman-Colman groups use of CuAAC active template synthesis to prepare carbazole-benzophenone-based rotaxanes that display thermally activated delayed fluorescence (TADF).^204^ A large increase in photoluminescence quantum yield results from threading one or two rings around the TADF emitting unit. The interlocked structure also increases photostability of the emitters and altered their emission wavelength, shifting it slightly to the red compared to the non-interlocked thread. These properties arise from the close proximity between the macrocycle and axle and resulting weak interactions that finely tune the HOMO energy level, the singlet–triplet energy gap, and the energy gap between the charge transfer states of the emitters.

A [2]rotaxane system capable of reversible electronic energy transfer (REET) between chromophores attached to the axle and macrocycle of a rotaxane has been reported by the McClenaghan and Goldup groups.^205^ Delayed luminescence and long luminescence lifetimes result from the close proximity of the chromophores.

Interlocked systems accessible through active template synthesis have also been investigated as single-ion magnets. The rigid coordination environment present in compact rotaxanes was used to design cobalt(ii) ligands that form complexes that show magnetic anisotropy and field-induced single-ion magnet behaviour.^206^

The restriction of interlocked component (co-)conformational freedom in relatively compact rotaxanes accessible by active template synthesis can manifest itself in effects observable by EPR^207^ and/or NMR spectroscopy.^208^ The restricted dynamics influences EPR resolution^207^ and hydrogen bonding interactions^208^ can be directly probed through ^1^H–^15^N multiple quantum coherence NMR experiments.

### Oligomers, polymers and other materials

**Fig. 45:**
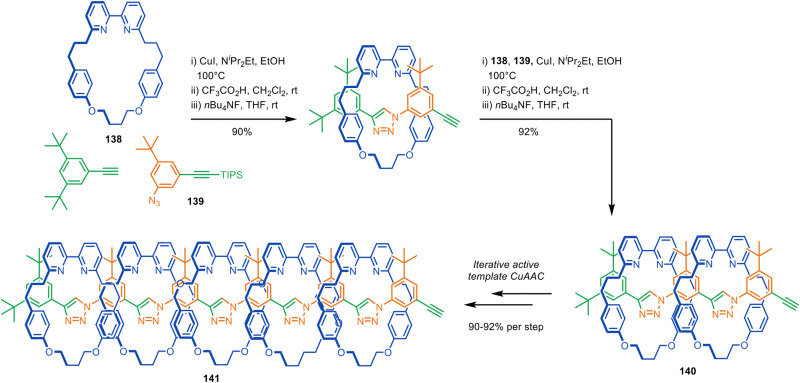
Goldup's synthesis of homo[6]rotaxane 134 through iterative high-yielding active template CuAAC reactions.^210^

Mechanically interlocked polymers are appealing materials due to the structural stability conferred by the mechanical bond and their potential for stimuli-responsive behaviour.^209^ Most poly[*n*]rotaxanes are prepared under thermodynamic control (*i.e.* conventional passive template synthesis) or by the polymerisation of pre-formed rotaxane monomers. However, Goldup and co-workers have shown that well-defined oligo[*n*]rotaxanes can be prepared through iterative CuAAC active template synthesis (Fig. 45).^210^ A series of monodispersed rotaxane oligomers of controlled length was prepared using small bipyridine macrocycles, such as 138, and functionalised monomer 139 in a series of sequential coupling and deprotection steps. Homo[*n*]rotaxanes such as 140 and 141, as well as hetero[*n*]rotaxanes incorporating different macrocycles threaded in sequence, were synthesised in high overall yield (90–92% per step). The reduced reactivity of sequential active template reactions around a branched central unit due to the steric bulk of incoming macrocycles was later harnessed for the stepwise synthesis of [3]- and [4]rotaxanes from a 1,3,5-triethynylbenzene core.^41^

The Goldup and Tavassoli groups have prepared oligonucleotide-based rotaxanes by adapting click DNA ligation^211,212^ to active template CuAAC synthesis.^213^ Using a bipyridine macrocycle and oligonucleotide strands functionalised with an alkyne or azide group in the 3′ and 5′ positions in their chain termini, two different mechanically interlocked DNA strands could be prepared in good yield. The threaded macrocycle had a shielding effect on duplex formation, inhibiting DNA hybridisation and PCR amplification of the oligonucleotide rotaxanes compared to non-interlocked analogues.

Hybrid organic–inorganic interlocked structures offer an alternative class of materials. Szyszko and co-workers have used CuAAC active template synthesis for the assembly of a series of hybrid organic–inorganic rotaxanes incorporating cubic silesquioxanes as one of the stoppering elements.^214^ The hybrid rotaxanes demonstrated improved thermal stability in the solid state compared to wholly organic analogues.

Mullen and co-workers have reported the CuAAC active template synthesis of [2]rotaxanes on polymer resins.^215^ This generated higher yields of interlocked products than comparable passive template solid phase synthesis.

### Molecular machinery

**Fig. 46:**
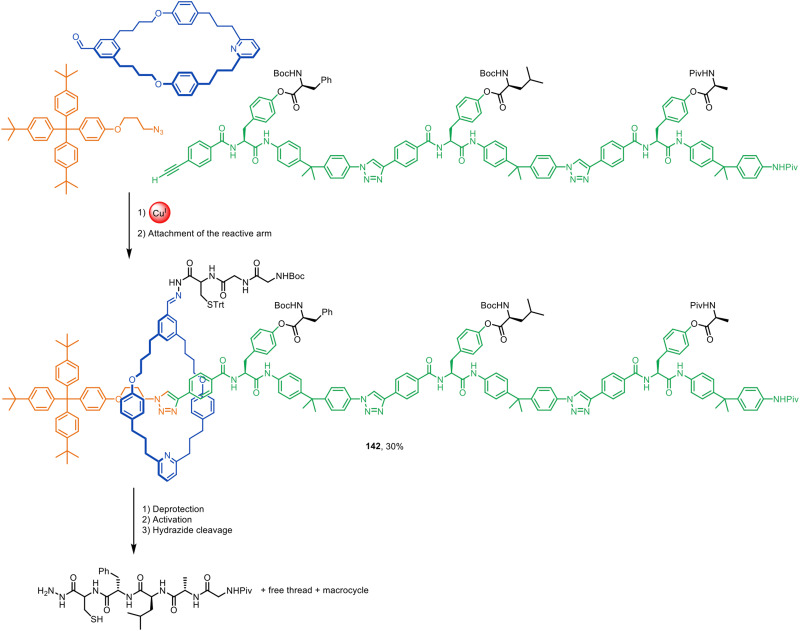
A rotaxane-based peptide synthesiser 135, assembled through CuAAC active template synthesis.^216^

The well-defined large amplitude dynamics possible in rotaxane and catenane architectures, together with active template synthesis being under kinetic control, has led to its exploitation in both the synthesis and operation of artificial molecular machines. Leigh and co-workers have used CuAAC active template synthesis to assemble rotaxane machines (*e.g.*142) that perform sequence-selective synthesis in a manner reminiscent of the ribosome (Fig. 46).^216–221^ The rotaxane design features a catalyst-bearing macrocycle that moves along the threaded axle, removing the amino acid barriers that block its path, transferring them to the growing peptide chain in the sequence it encounters them along the track (Fig. 46). Active template synthesis was also used to make a rotaxane synthon that could be added to longer tracks, resulting in higher overall yields than the original strategy.^217^ The concept was later extended to connect other types of building blocks in sequence to form oligomers and polymers, including nonproteinogenic β^3^-amino acids,^218^ leucine-ester-derivatised polystyrene chains,^219^ and oligomers joined through iterative carbon–carbon bond formation.^220^ Parallel operation of two molecular peptide synthesisers in the same pot was used to assemble a decapeptide.^221^

**Fig. 47:**
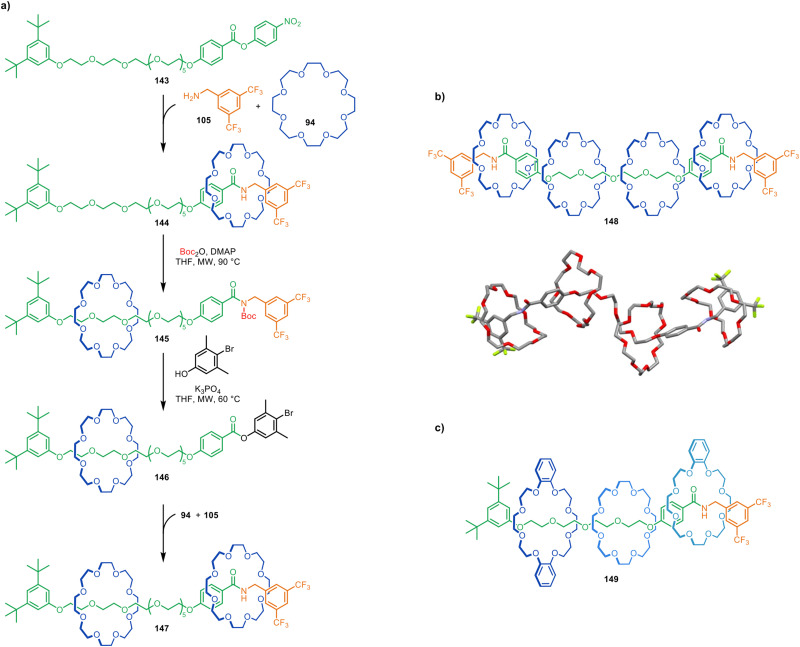
(a) A stepwise-operated molecular pump that makes use of successive metal-free active template transamidation reactions.^222^ Macrocycles are pumped onto the axles by a sequence of: (1) an active template transamidation reaction, (2) Boc-addition, and (3) substitution by an activated ester. (b) [5]Rotaxane 148 formed after two pumping cycles from a thread with pump motifs at either terminus. X-Ray crystal structure of 148. (c) [4]Rotaxane 149, in which the macrocycles have been threaded onto the axle in a controlled sequence.

Catalysis-driven metal-free active template synthesis has been used to demonstrate the mechanism of chemically fuelled molecular pumps. Molecular pump 143 contains an activated ester electrophile that causes 24-crown-8 (94) to thread through its accelerated reaction with benzylamine 105, forming amide rotaxane 144 (Fig. 47).^222^ Subsequent addition of a bulky *N*-Boc group forms 145, with the macrocycle shuttling to the oligo(ethylene glycol) collection thread. Substitution of the amide to form phenolic ester 146, followed by another crown ether threading active template reaction, generates [3]rotaxane 147.

Every pumping cycle can add an additional macrocycle to the thread for each activated ester present, and so [5]rotaxane 148 is obtained after only two transamidation pumping cycles from a thread with pumping modules at both ends. In this high energy, kinetically locked, structure an array of weak CH–O hydrogen bonds between the components is apparent from X-ray crystallography (Fig. 47b). By carrying out pumping cycles with different crown ether derivatives, it was also possible to synthesise rotaxanes with specific sequences of threaded macrocycles, such as 149 (Fig. 47c).

**Fig. 48:**
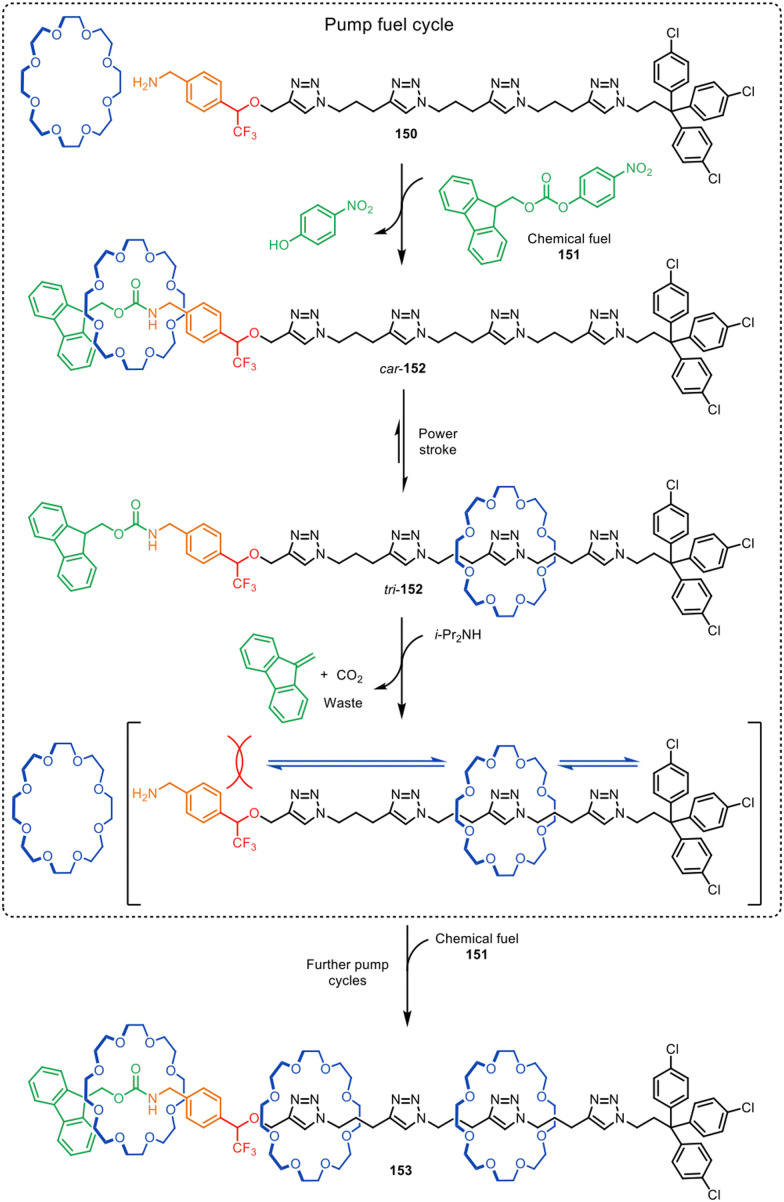
An autonomous catalysis-driven artificial molecular pump 150 which operates continuously in the presence of chemical fuel 151.^223^

Metal-free active template synthesis also enables the design of molecular pumps that operate autonomously, in which macrocycles are continuously pumped onto an axle, away from a state of equilibrium, powered by a chemical fuel.^223^ Catalysis-driven molecular pump 150 features a free benzylic amine at one end and a bulky stopper at the other (Fig. 48). The poor binding affinity of crown ether macrocycles for triazoles means that a vanishingly small proportion of macrocycles are threaded on the axle at equilibrium. However, an Fmoc-derived chemical fuel, 151, acts as an electrophile for a crown ether metal-free active template reaction, forming [2]rotaxane *car*-152 (the italicised prefix refers to the position of the ring on the axle). The Fmoc-carbamate group prevents macrocycle dethreading and the restricted steric environment around the carbamate site results in the macrocycle being displaced to the catchment region of the thread (*tri*-152). The presence of i-Pr_2_NH base in the reaction mixture triggers decomposition of the Fmoc-carbamate group faster when the bulky macrocycle is distant from the carbamate group. The trifluoromethyl group (shown in red in Fig. 48) is sufficiently large (a molecular ‘speed bump’) that it slows dethreading of the macrocycle from the catchment region off the open amine-terminated end of the thread.^224^ Therefore pumping of another crown ether onto the axle occurs faster than dethreading of the ring already on the axle, leading to the formation of [4]rotaxane 153. Macrocycles are thus continuously pumped onto the axle until all of the Fmoc-fuel is depleted, after which the rings dethread to regenerate the original ring equilibrium distribution in solution.

### Unusual reactivity and mechanistic insights

As noted earlier, active template synthesis can provide mechanistic information about the catalytic manifolds employed, for example when more than one ring is unexpectedly threaded onto an axle or multiple strands are encircled by a single ring.^28,36,39,40,48,61,102,103^ Sometimes the mechanical bond can lead to other unexpected reaction outcomes. Chaplin *et al.* reported the selective formation of *E*-enyne products in their Rh(i)-mediated active template formation of pseudorotaxanes (Fig. 21)^117^ and have observed unusual C–C σ-bond oxidative additions^118^ (see section on zinc-, cobalt-, ruthenium- and rhodium-mediated active template synthesis).

**Fig. 49:**
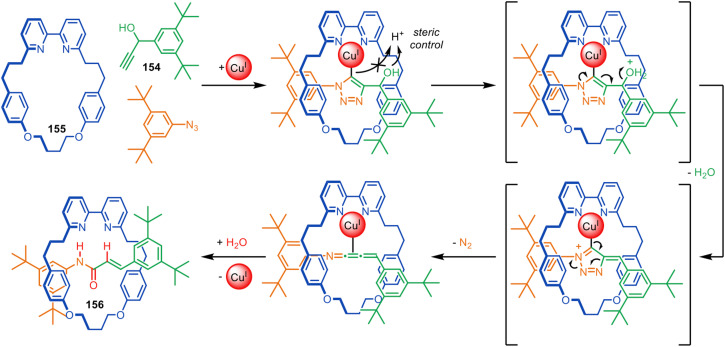
Proposed mechanism of the tandem active template CuAAC rearrangement reported by Goldup and co-workers.^225^

Goldup and co-workers found an unusual tandem process occurred during the active template CuAAC synthesis of [2]rotaxanes involving propargylic alcohol 154 as the alkyne substrate. When small bipyridine macrocycle 155 was used, a post-assembly rearrangement of the stabilised Cu(i)-triazolide interlocked derivative was triggered rendering the unexpected acrylamide rotaxane 156 (Fig. 49).^225^ Mechanistic studies suggest that mechanical bond stabilisation of the triazolide intermediate and the steric constraints imposed during rotaxane formation alters the chemoselectivity of the metal-catalysed reaction.

## Conclusions

The active template synthesis of entwined and interlocked structures is an efficacious and versatile strategy that complements conventional passive template synthesis. In addition to often requiring only sub-stoichiometric quantities of metal salts (or no reagents whatsoever in the case of metal-free active template synthesis), other key advantages include that its use can be traceless, without the need for binding sites in both components to be threaded or interlocked. Furthermore, the assembly process is carried out under kinetic control, enabling the synthesis of interlocked products that are not necessarily thermodynamically stable. Accordingly, threaded structures can be generated with functional groups held in close proximity to each other in (co)-conformations that might not be accessible through other synthetic strategies. Active template synthesis often proceeds in high yields, with simple experimental procedures and readily available starting materials. Rotaxanes formed through active template chemistries are currently being investigated for potential applications in fields as diverse as sensing, catalysis, drug delivery, materials and molecular machinery.

Much of the early work in this field focussed on expanding the toolbox of catalytic reactions suitable for active template synthesis. A particularly attractive target remains finding a way to maintain the coordination of Pd(0) to a component throughout the catalytic cycle so that Pd(0)-mediated cross-coupling reactions can be used. Other challenges arise from functional group tolerance, and the ligand requirements of particular metal-catalysed reactions for active template synthesis if the preparation or handling of the building blocks is problematic.

Finally, while substantial progress has been made in the synthesis of interlocked molecules over the last four decades, practical applications of rotaxanes and catenanes remain scarce. The lack of requirements for permanent binding motifs may make active template synthesis more amenable for making practically useful interlocked structures than other synthetic strategies. Active template synthesis appears a promising route to rotaxanes and catenanes with applications in nano-electronics and biotechnology. Further exploration of different metal-mediated and metal-free active template methodologies, coupled with potential applications of newly accessible structure types, augurs well for the continued advancement^18^ of the chemistry of mechanically interlocked molecules.

## Author contributions

This Review was written through the contributions of all authors.

## Data availability

No primary research results, software or code have been included and no new data were generated or analysed as part of this review.

## Conflicts of interest

There are no conflicts of interests to declare.
